# Highly selective hydrogenation of arenes using nanostructured ruthenium catalysts modified with a carbon–nitrogen matrix

**DOI:** 10.1038/ncomms11326

**Published:** 2016-04-26

**Authors:** Xinjiang Cui, Annette-Enrica Surkus, Kathrin Junge, Christoph Topf, Jörg Radnik, Carsten Kreyenschulte, Matthias Beller

**Affiliations:** 1 Leibniz-Institute for Catalysis, University of Rostock, Albert Einstein Street, 29a, Rostock 18059, Germany

## Abstract

Selective hydrogenations of (hetero)arenes represent essential processes in the chemical industry, especially for the production of polymer intermediates and a multitude of fine chemicals. Herein, we describe a new type of well-dispersed Ru nanoparticles supported on a nitrogen-doped carbon material obtained from ruthenium chloride and dicyanamide in a facile and scalable method. These novel catalysts are stable and display both excellent activity and selectivity in the hydrogenation of aromatic ethers, phenols as well as other functionalized substrates to the corresponding alicyclic reaction products. Furthermore, reduction of the aromatic core is preferred over hydrogenolysis of the C–O bond in the case of ether substrates. The selective hydrogenation of biomass-derived arenes, such as lignin building blocks, plays a pivotal role in the exploitation of novel sustainable feedstocks for chemical production and represents a notoriously difficult transformation up to now.

## Abstract (web summary)

The selective reduction of arenes is important in organic synthesis and also valorization of biomass. Here, the authors report the use of ruthenium-based nanoparticles, which display high activity in arene reduction and preferentially hydrogenate aromatic rings rather than cleaving etheric C-O bonds.

## Graphical abstract

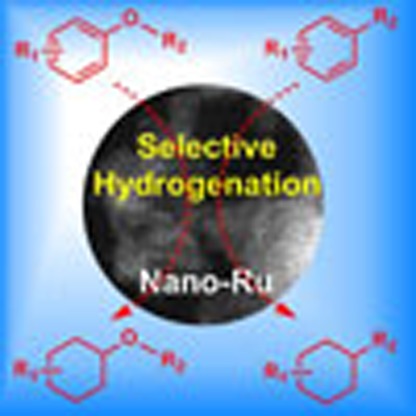

Catalytic hydrogenations constitute economic and clean transformations for many pharmaceutical and petrochemical processes. Due to the low price of the reductant and the 100% atom efficiency of the overall reaction, they adopt a privileged position in methodological tool box of the chemical industry. In the future, this type of transformation is expected to become even more interesting and essential because of the increasing importance of the valorization of oxygen-rich biomass. Till now, the vast majority of industrially produced chemicals depend on fossil raw materials namely petroleum, natural gas and coal. Hence, the conversion of renewable biomass to higher value-added platform chemicals currently attracts considerable attention^1^,^2^,^3^,^4^. In this respect, significant research activities are focusing on the valorization of lignin, which is one of the most abundant available feedstocks^5^,^6^. After lignin depolymerization, highly oxygenated aromatic monomers are obtained. For the consecutive utilization of such lignin-derived compounds, both heterogeneous materials and homogeneous catalysts based on Pd^7^,^8^,^9^, Pt^10^,^11^,^12^, Ru^13^,^14^,^15^, Rh^16^ and Ni^17^,^18^,^19^,^20^,^21^,^22^,^23^,^24^,^25^,^26^,^27^,^28^,^29^ have been developed which allow for reductive C–O cleavage reactions that produce alcohols or alkene bio-building blocks (Fig. 1). Apart from this general approach, we believe it is highly desirable to develop alternative strategies based on selective arene hydrogenation to effectively utilize lignin-derived compounds and other oxygenated (hetero)arenes as feedstock for both bulk and fine chemical production.

In general, selective hydrogenation of aromatic rings plays an important role in the generation of all kinds of aliphatic derivatives, which are crucial starting materials in synthesis of polymers, resins, dyes and fine chemicals. Hence, this catalytic transformation represents also an attractive candidate for the investigation of the selective hydrogenation of lignin-derived fragments. The resulting alicyclic ethers constitute a class of very promising intermediates in the production of fine chemicals and bio-fuels^9^,^30^,^31^. Although hydrogenations of the arene rings of lignin building blocks are well-known, reports on the development of catalysts for the selective generation of the alicyclic ethers from such compounds are scarce^16^.

Nowadays, heterogeneous ruthenium nanoparticles (Ru-NPs) represent state-of-the-art catalysts for selective hydrogenation of aromatic rings which are also lower in price (4,200$ ozt^−1^) compared with other noble metals (for example, Pd: 60,450$ ozt^−1^). By tuning shape and size of the NPs, type of supports and even by adding functionalized ligands, the performance of these materials has been greatly improved^32^,^33^,^34^,^35^,^36^,^37^,^38^. Despite all these efforts, the reported systems exhibit only low selectivity for the hydrogenation of highly reactive benzylic ethers and related derivatives. On the basis of our recent work on the development of metal NPs modified with nitrogen-doped graphene layers (NGrs)^39^,^40^,^41^,^42^,^43^,^44^,^45^,^46^, we thought that such materials might allow for more selective hydrogenations. The incorporation of nitrogen atoms into a carbon matrix has been proven to affect the catalytic activity/selectivity of the resulting materials tremendously^47^,^48^. Hence, many efforts have been devoted in recent years to the development of more active *N*-doped carbon materials including their usage as supports in catalysis^49^,^50^,^51^,^52^. As an instructive example in the context of lignin valorization, a Pd@CN_0.132_ catalyst was prepared and successfully applied in the hydrogenation of vanillin^53^.

Here, we describe the preparation, characterization and catalytic testing of novel ruthenium-based NP immobilized on a *N*-doped carbon support. The resulting optimal catalyst allows for unique hydrogenation of all kinds of substituted arenes including lignin-derived aromatic compounds to give the aliphatic congeners in both high activity and selectivity.

## Results

### Material preparation and characterization

At the start of our work, we synthesized different Ru-NPs immobilized on Vulcan powder (Ru@NDCs-X; X labels the pyrolysis temperature). To incorporate nitrogen atoms mainly three different sources (dicyanamide (DCA), cyanamide and phenanthroline) were used (Supplementary Fig. 1). The preparation of these catalysts commenced with the impregnation of Vulcan powder with an ethanolic solution of a nitrogen-ligated RuCl_3_ complex. The Ru@NDC nano-composites were obtained upon solvent evaporation and subsequent pyrolysis at 600, 800 or 900 °C under inert conditions. Preliminary screening of all the different catalysts revealed best results for the DCA-based materials (Table 1). Applying DCA as ligand elemental analysis of the resulting catalysts indicated that the nitrogen content dropped from 0.65 to 0.4 wt% as the pyrolysis temperature is increased from 600 to 900 °C (Supplementary Table 1 and Fig. 3). These findings are in agreement with the results obtained by XPS. Further evidence of *N*-doping is provided by X-ray photoelectron spectroscopy (XPS). As shown in Fig. 2e and Supplementary Fig. 5, three types of nitrogen were found for the samples pyrolysed at 600, 700 and 800 °C, namely: pyridinic, pyrrolic and NO_x_ species, respectively. For the sample heat-treated at 600 °C, the amount of pyrrolic N was found to be only marginally higher than the pyridinic congener (49.7 versus 41.2%), whereas in the sample pyrolysed at 700 °C the conditions moved into reverse (37.4 versus 55.8%). For the 800 °C sample, the pyrrolic N was found to be the dominating species again (78.0 versus 12.3%). The quantitative analysis revealed a near-surface amount of 1.7 at% N after treatment at 600 °C and 700 °C, which decreased slightly to 1.4 at% at 800 °C. To better investigate the effect of doped-nitrogen, we synthesized Ru@NDCs-800 with different nitrogen content by adjusting the amount of DCA in the synthetic process. Interestingly, decreasing the ligand amount to 100 mg, the N content decreased to 0.66 at%, whereas no obvious change was observed for the catalyst prepared using 400 mg of ligand (Supplementary Table 3). The amount of N dropped significantly to 0.5 at% after treatment at 900 °C. This low nitrogen content hampered the determination of the various modifications of the nitrogen. On the basis of XPS analyses, and in agreement with other NP immobilized on N-doped carbon supports^54^, we tentatively assign the corresponding signals to pyridinic and graphitic N, respectively. Due to the influence of the content and valence of metal-based species on the heterogeneous hydrogenation, Ru 3p XPS analyses of Ru@NDCs-catalysts were measured. These analyses suggest the formation of a RuO_2_ phase and interaction of the active Ru sites with nitrogen species (Supplementary Fig. 4)^55^. To gain more insight into the morphology and structure of Ru@NDCs-800, high-resolution transmission electron microscopy analysis of the material was performed. The Ru-NPs are finely dispersed on the support and the average size of the NPs amount to 1.82±0.11 nm (Fig. 2a). On raising the pyrolysis temperature to 900 °C, the particle size of the resulting material increased to 2.5–3.0 nm (see Supplementary Fig. 2). However, in the absence of DCA the Ru-NPs aggregated to form larger entities ranging from 10 to 20 nm particles size. These results revealed that the Ru-NPs were prevented from aggregation upon decomposition of DCA and concomitant N incorporation into the carbon matrix during the course of the pyrolysis process. In addition, the formed Ru-NPs might be stabilized by the pyridinic N and pyrrolic N because their lone-pair electrons can serve as metal coordination sites^56^. Figure 2b shows the images of the crystal plane of Ru (101), and the corresponding plane spacing was found to be 0.2 nm. This result is in good agreement with the value obtained by X-ray diffraction (Supplementary Fig. 6). As shown from the Figs 2c-2d, the Ru-based *N*-doped carbon material develops a pronounced, well-textured morphology upon heat treatment at 800 °C.

### Catalysis

After the initial screening, the performance of the prepared materials resulting from DCA was evaluated in more detail. Here, the hydrogenation of phenol to cyclohexanol with H_2_ pressures ranging from 5 bar to 20 bar was chosen as an industrially relevant benchmark reaction, which is of interest for bulk polyester processes^57^,^58^,^59^. We commenced our survey by control experiments with Ru-free carbon and specifically prepared the *N*-doped carbon, but in neither case any product formation was observed (Table 1, entries 1 and 2). A standard Ru/C catalyst obtained by pyrolysis at 800 °C in the absence of ligand (DCA) exhibited only moderate activity at 5 bar H_2_ and 40 °C (Table 1, entry 3). To our delight, the activity of the *N*-modified Ru@NDC-800 catalyst is considerably increased and an almost quantitative yield of cyclohexanol is achieved at low temperature and pressure (Table 1, entry 6). In contrast, the Ru@NDCs samples originating from lower pyrolysis temperature featured significantly lower reactivity (Table 1, entries 4, 5 and 7). When the reaction was carried out for 1 h, a significantly higher activity was also obtained in the presence of Ru@NDCs-800 (Supplementary Table 3). However, Ru@NDCs-600 or Ru@NDCs-700 might show higher TOFs based on active Ru atoms on the surface of the NPs. Moreover, the Ru@NDCs sample synthesized using low amount of ligand gave considerably lower conversion, whereas similar activity was obtained with the sample prepared in the presence of 400 mg of ligand (Table 1, entries 9 and 10). This also demonstrates the importance of a critical amount of nitrogen.These experimental findings demonstrate the importance of nitrogen for the activity of the catalyst. Next, the reusability of the Ru@NDCs-800 nano-composite was examined since catalyst recyclability represents an integral part in the economic assessment of chemical transformations. The active material was separated from the reaction mixture via centrifugation and reused directly for five times. Gratifyingly, no obvious deactivation was detected and the yield of the desired product amounted to 93% at the sixth cycle (Table 1, entries 6, 11–15).

### Selective hydrogenation of arenes

The functional group tolerance survey of various arene substrates was conducted with the Ru@NDCs-800 catalyst. As shown in Table 2, the hydrogenation of methyl benzoate was complete at room temperature within 12 h (Table 2, entry 1). Furthermore, benzamide and phthalimide underwent selective reduction retaining the amide and imide groups in the substrates (Table 2, entries 2 and 3). In case of quinoline, which is used as a feedstock in the production of specialty chemicals, the heterocyclic 1,2,3,4-tetrahydroquinoline is obtained in 87% yield, whereas selective hydrogenation of the pyridine ring occurred (Table 2, entry 4). Ru@NDCs-800 also exhibited good activity in the hydrogenation of methyl benzoylformate, affording a cyclohexyl-tagged tertiary alcohol in 81% yield (Table 2, entry 5). The hydrogenation of benzyl alcohol proceeded smoothly with almost quantitative formation of the corresponding saturated alicyclic alcohol. It should be noted that the yield of the latter product decreased to 75% when commercial Ru/C was used as catalyst. This lower yield is explained by concomitant C–O bond cleavage which leads to the formation of a considerable amount of methyl cyclohexane (Table 2, entry 6). *N*-ethylcarbazole is regarded as a promising future hydrogen-storage material^60^,^61^,^62^. Hence, we tested our Ru@NDCs-800 catalyst in the hydrogenation of this peculiar heterocycle. Interestingly, at 10 bar and 100 °C *N*-octahydroethylcarbazole is obtained in 85% yield, demonstrating the possibility of selective partial hydrogenation. Simply by increasing the temperature to 100 °C, the fully hydrogenated perhydro-derivative was isolated in 93% yield (Table 2, entries 7 and 8).

### Hydrogenation of benzylic compounds

In general, hydrogenolysis of benzylic compounds is observed in the course of hydrogenation processes. Especially, in benzylic ethers and alcohols the cleavage of the C–O bond easily occurrs due to the lower C–O bonding dissociation energy. For example, the bonding dissociation energy for benzylic ethers is 220 kJ mol^−1^ compared with 310 kJ mol^−1^ for biaryl ethers and 290 kJ mol^−1^ for arylethyl ethers^27^. Indeed, in the hydrogenation of benzyl phenyl ether using commercial Ru/C partial cleavage of the benzylic C–O linkage is observed. Hence, a mixture of products including the fully hydrogenated ether, cyclohexanol and methyl cyclohexane is obtained (Fig. 3, **1a**). However, applying the novel Ru@NDCs-800 catalyst gave excellent selectivity for the hydrogenation of the arene rings. As shown in Table 3, benzyl phenyl ether was smoothly converted to the corresponding alicyclic ether in 93% yield. Even at 50 bars of hydrogen no significant C–O cleavage is observed. Similarly, substrates with pendant functional groups on the phenyl moiety such as hydroxyl, methyl and even long alkyl chains were well-tolerated and gave high yields of the corresponding *cis*/*trans* ethers (ratio 1.5–4:1; Fig. 3, **1b–1d**). In addition, 1-methyl-4-(phenoxymethyl)benzene was also converted to the corresponding ether in 78% yield (Fig. 3, **1e**). All these results demonstrate the specific behaviour of *N*-doped ruthenium nano-composites for selective hydrogenation of aromatic rings in the presence of the benzylic C–O bonds.

**Figure 1:**
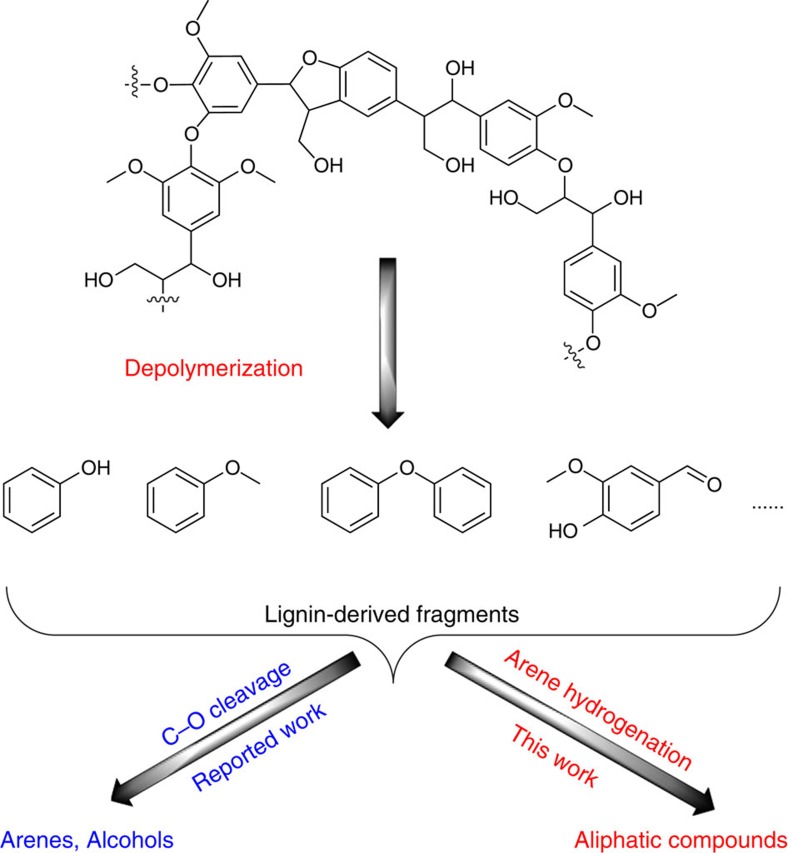
Valorization of lignin-derived building blocks. A new route demonstrated.

**Figure 2:**
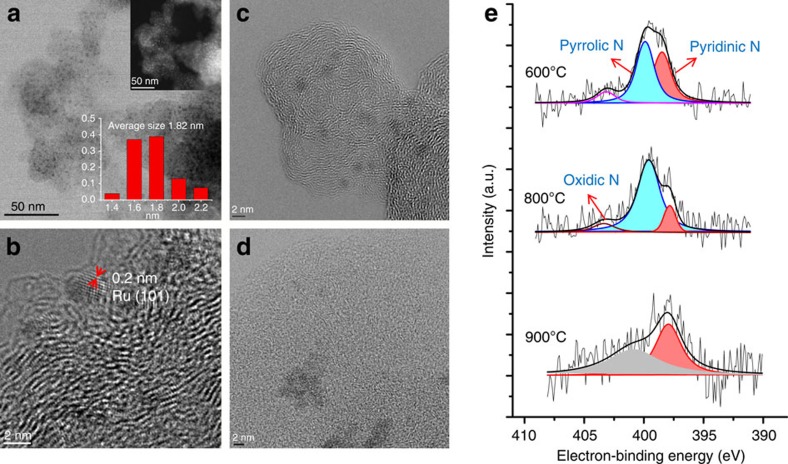
High-resolution transmission electron microscopy images. Bright field HRSTEM images (**a**,**c**) and HRTEM (**b**) of the Ru@NDCs-800 (inset of **a**: HAADF image), the HRTEM image (**d**) of the Ru@C-800 and N1s XPS spectrum (**e**) of Ru@NDCs.

**Figure 3:**
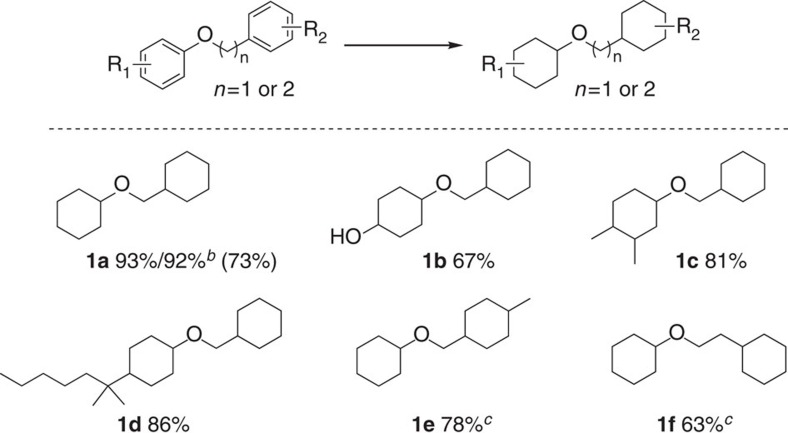
Selective hydrogenation of benzylic ethers using Ru@NDCs-800. Reaction conditions: 0.5 mmol substrate, 20 mg catalyst, 2 ml isopropanol, 20 bar H_2_, 60 °C, 24 h. Isolated yields, value in parentheses is obtained using commercial 5% Ru/C. ^*b*^50 bar H_2_. ^*c*^GC yield.

As pointed out *vide supra* various phenethyl ethers are easily available as platform chemicals from abundant lignin. As an example for this class of compounds the hydrogenation of phenethyl ether was investigated. Gratifyingly, the Ru@NDCs-800 showed good activity for arene hydrogenation of phenethoxybenzene (Fig. 3, **1f**).

### Hydrogenation of aromatic ethers

To expand the scope of this arene hydrogenation, next we investigated the reactivity of various alkyl aryl ethers. Anisole, ethoxybenzene, butoxybenzene and (octyloxy)benzene were readily converted to the corresponding products in yields ranging from 88–94% (Table 3, entries 1–4). Further alkyl and methoxy substituents had no negative impact on the catalytic activity and the fully hydrogenated products were obtained as *cis*/*trans* regioisomers (3–9:1; Table 3, entries 5–8). Arene hydrogenation of 1-methoxynaphthalene was also realized with 90% of the desired ethers formed. In addition, 2,3-dihydrobenzofuran and benzofuran were converted to the corresponding ethers in 84–87% yield. Notably, tetrahydrofurfuryl alcohol is obtained by selective hydrogenation of furfuryl alcohol, which is manufactured industrially. Moreover, the successful hydrogenation of 4-propylguaiacol and vanillin, which are both directly accessible from lignin depolymerization, was demonstrated and good yields were achieved (Table 3, entries 13 and 14). Having verified the catalytic activity of Ru@NDCs-800 in the selective hydrogenation of alkyl aryl ethers, we tested the established catalytic protocol in the transformation of lignin-derived biphenyl ethers.

As shown in Table 4, the conversion of a broad range of substituted biaryl ethers into the corresponding aliphatic ethers proceeded smoothly. The parent compound afforded the corresponding ether in 84% yield, whereas the hydrogenation of alkyl-substituted derivatives provided slightly higher product yields ranging from 85 to 89% (Table 4, entries 1–4).

Performing the latter reaction in the presence of commercial 5% Ru/C, the yield for the desired product decreased to 65% due to C–O cleavage. Finally, dibenzo-fused five- and six-membered heterocyclic substrates displayed similar reactivity and the yield of the corresponding saturated tricyclic ethers amounted to 81–91% (Table 4, entries 5–8). To investigate the high selectivity of the catalytic system, the hydrogenolysis of aliphatic ethers such as methoxycyclohexane and oxydicyclohexane were tested and no C–O cleavage occurred. This result revealed that hydrogenation of aromatic rings is favoured compared with the cleavage of C–O bonds. The experimental findings were also substantiated by variation of the solvent (see Supplementary Table 2), whereas higher selectivity was obtained using cyclohexane as reaction medium by virtue of its property to inhibit the hydrogenolysis reaction.

## Discussion

In summary, we developed for the first time a new type of ruthenium nano-composite immobilized on a carbon support. The described material contains finely dispersed ruthenium, which is in contact with a specific carbon–nitrogen matrix. The resulting catalysts (Ru@NDCs) are easily obtained in a practical and scalable two-step method via pyrolysis of simple ruthenium trichloride and inexpensive DCA. Combination of different analytic methods including XPS revealed the formation of graphitic nitrogen, which is identified as the functional prerequisite for the development of catalytic activity. The optimal catalyst exhibited good-to-excellent activity in the selective hydrogenation of arenes, particularly in the transformation of aromatic ethers to the corresponding alicyclic compounds with preservation of the phenyl- and benzyl C–O bonds. The utility of the catalyst opens new avenues for the valorization of lignin-derived aromatic compounds to provide novel sustainable platform chemicals. In addition, industrially relevant processes such as the hydrogenation of phenol proceed under mild conditions in a green manner.

## Methods

### General

Unless otherwise specified, reagents and solvents were purchased from Aldrich, Fluka, Acros and Strem commercially and used as received. All compounds were characterized by ^1^H NMR, ^13^C NMR, GC-MS spectroscopy. ^1^H and ^13^C NMR spectra were recorded on Bruker Avance 300 (300 MHz) or 400 (400 MHz) NMR spectrometers. The ^1^H and ^13^C NMR chemical shifts are reported relative to the centre of solvent resonance (CDCl_3_: 7.26 (1H), 77.16 (13C)). EI mass spectra were recorded on an MAT 95XP spectrometer (70 eV, Thermo Electron Corporation). For GC analysis, HP 6890 chromatograph with a 29 m HP5 column was used. GC-MS analysis was conducted on an Agilent GC-MS-HP5890 instrument. The products were isolated from the reaction mixture by solvent evaporation.

### Typical preparation of Ru@NDCs-catalysts

RuCl_3_ (0.5 mmol) and DCA (200 mg) were dissolved in ethanol and stirred for 2 h at 60 °C. Then, carbon support (VULCAN XC72R, ordered from PT (Cabot Indonesia) was added and the suspension was stirred at 60 °C for a further period of 2 h. After that, the mixture was cooled to room temperature and dried *in vacuo* at 60 °C for 2 h, and it was grinded to a fine powder which was subsequently pyrolyzed at 600, 700, 800 or 900 °C for 2 h under an argon atmosphere.

### Catalytic hydrogenation of phenol

In a 4 ml reaction vial equipped with a magnetic stirring bar, phenol (0.5 mmol) was mixed with 2 ml IPA. Then 20 mg of the ruthenium-based catalyst (20 mg) was added. The reaction vials were fitted with cap and needle and then placed into a 300-ml autoclave. The autoclave was purged thrice with H_2_ (10 bar), pressurized to 5 bar H_2_, placed into an aluminium block, heated to 40 °C and the reaction vessels were stirred for 2 h. After completion of the reaction, the autoclave was cooled to room temperature, *n*-dodecane was added to the reaction mixture as external standard and the mixture was diluted with ethyl acetate (20 ml), followed by filtration and analysis of a sample by GC and GC-MS. The crude reaction mixture was concentrated *in vacuo* and the obtained product was analysis by NMR.

For NMR analysis of the compounds in this article, see Supplementary Methods and Supplementary Figs 7–62.

## Additional information

**How to cite this article:** Cui, X. *et al*. Highly selective hydrogenation of arenes using nanostructured ruthenium catalysts modified with a carbon–nitrogen matrix. *Nat. Commun.* 7:11326 doi: 10.1038/ncomms11326 (2016).

## Supplementary Material

Supplementary Information Supplementary Figures 1-62, Supplementary Tables 1-3 and Supplementary Methods

## Figures and Tables

**Table 1:**
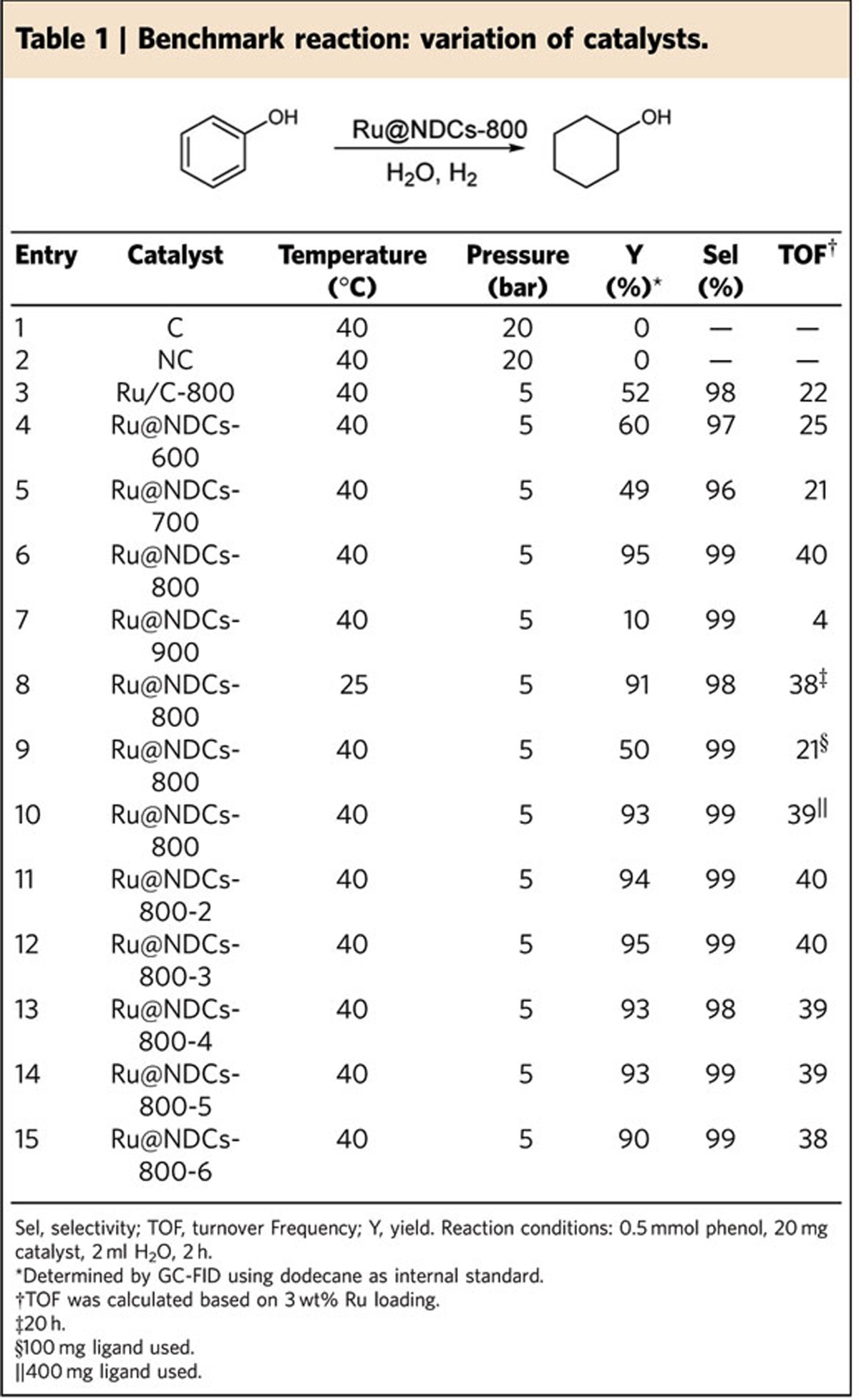
Benchmark reaction: variation of catalysts.

**Table 2:**
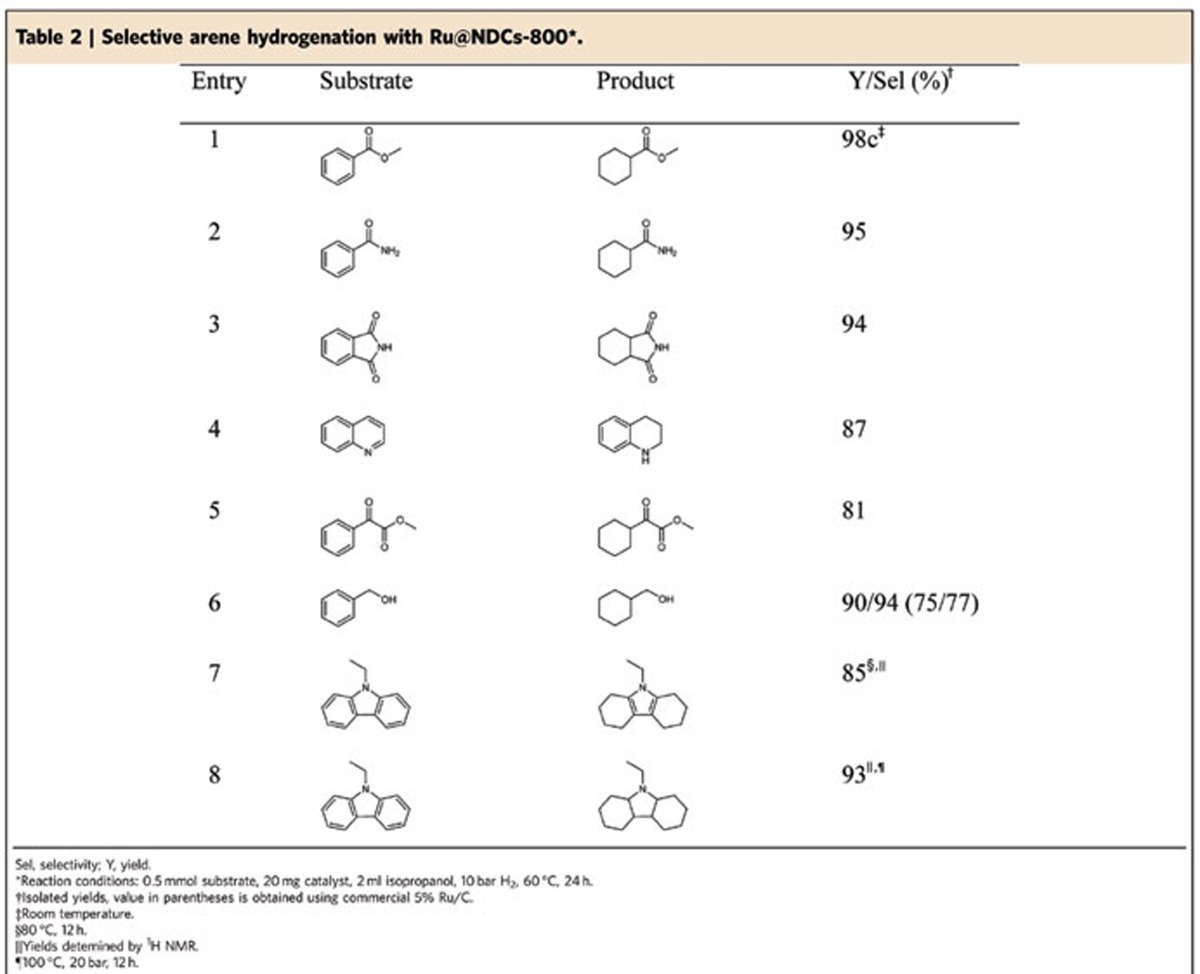
Selective arene hydrogenation with Ru@NDCs-800^*^.

**Table 3:**
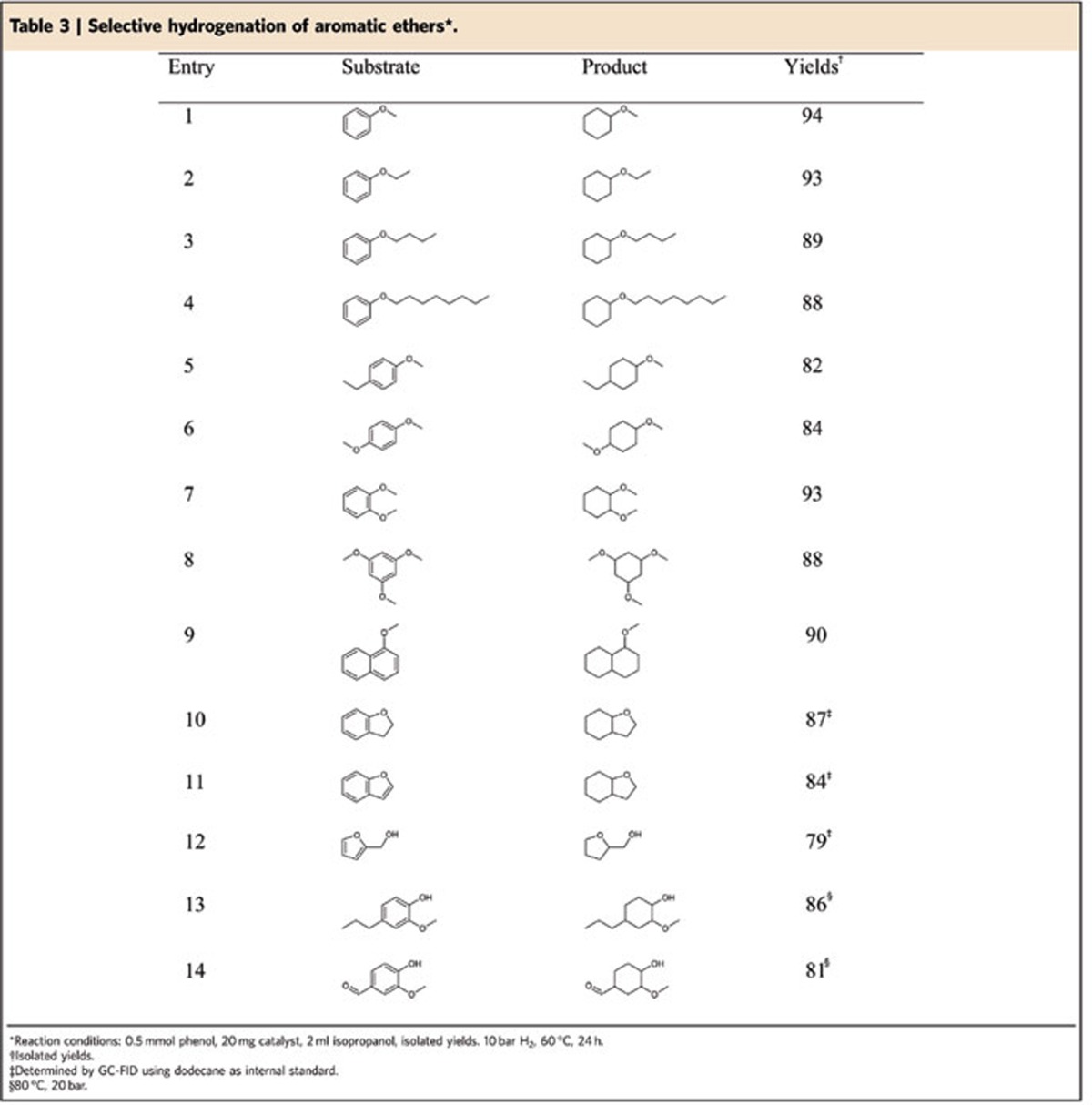
Selective hydrogenation of aromatic ethers^*^.

**Table 4:**
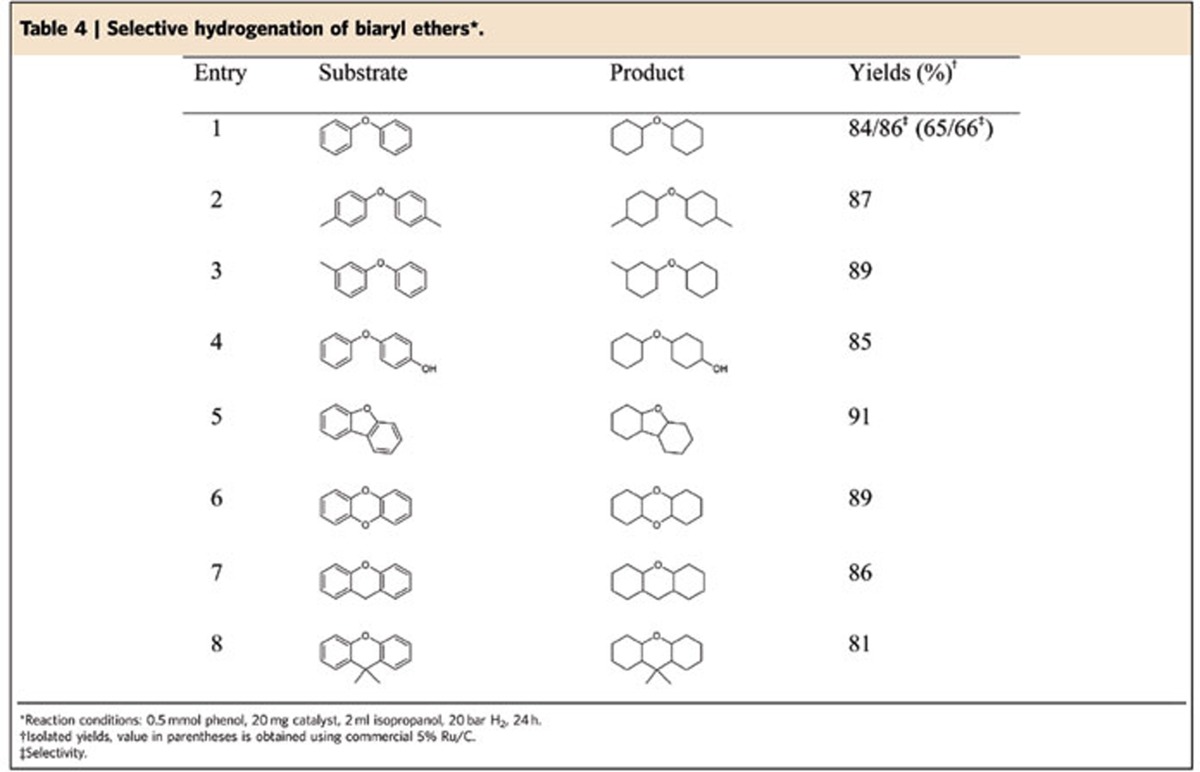
Selective hydrogenation of biaryl ethers^*^.
