# Supplementary material for: Highly selective hydrogenation of arenes using nanostructured ruthenium catalysts modified with a carbon–nitrogen matrix
Source: Nat Commun. 2016 Apr 26;7:11326. doi: 10.1038/ncomms11326 (PMC4853427; doi:10.1038/ncomms11326)
Supplement: Supplementary Information — Supplementary Figures 1-62, Supplementary Tables 1-3 and Supplementary Methods [file ncomms11326-s1.pdf]

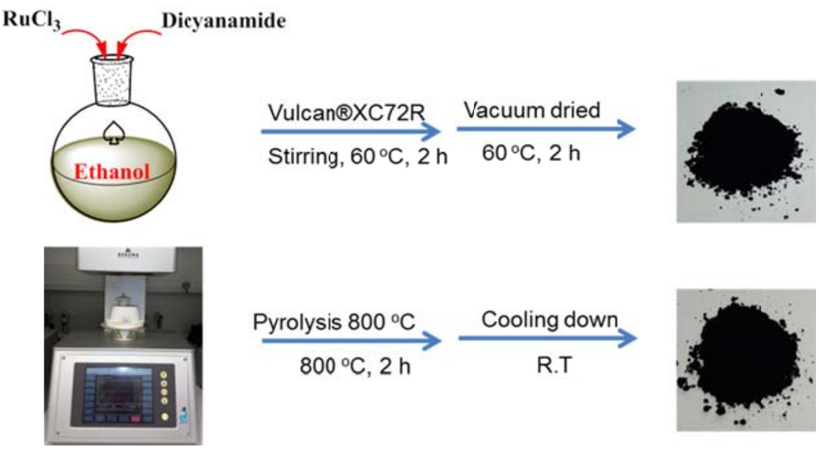

Supplementary Figure 1 Preparation of catalysts.

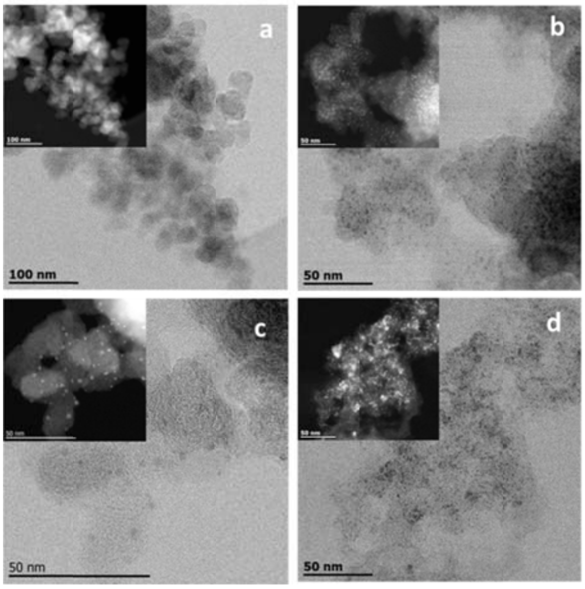

Supplementary Figure 2, HRSTEM of the Ru@NDCs-600 (a), Ru@NDCs-800 (b), Ru@NDCs-900 (c) and Ru@C-800 (d) (inset: HAADF images).

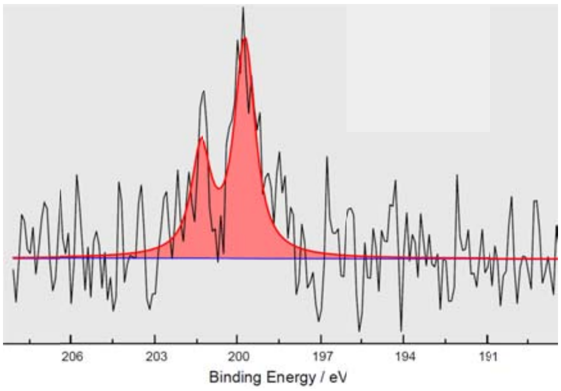

Supplementary Figure 3, Cl_{2p} XPS analysis of the Ru@C-800 prepared in the absence of dicyanamide.

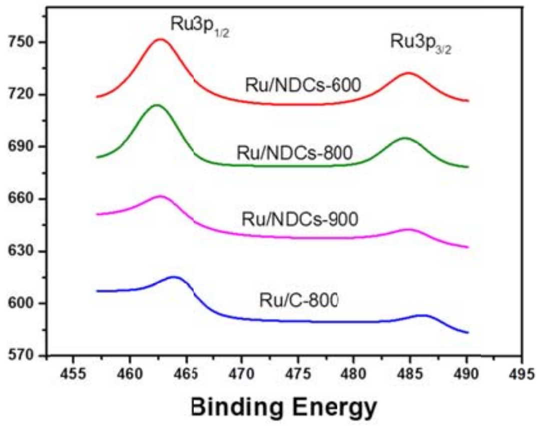

Supplementary Figure 4, Ru_{3p} XPS analysis of the prepared catalysts.

The Ru 3p XPS analysis of Ru@NDCs-600 and Ru@NDCs-800 showed a major peak at 642.5 eV (Figure S4), which is likely attributed to the RuO_2 phase. In the case of Ru@NDCs -900, the bonding energy for Ru 3p was shifted to 642.7 eV. However, these bonding energies for Ru@NDCs-catalysts appeared at lower binding energy compared to that of Ru-C prepared at ligand free condition (643.9 eV), suggesting that the incorporated nitrogen atoms donated electron density into the Ru active sites.

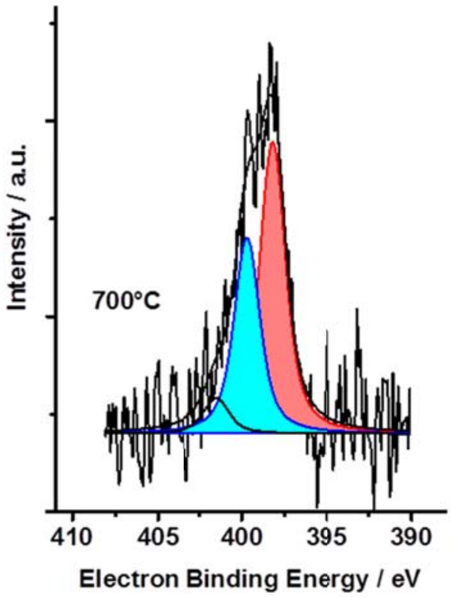

Supplementary Figure 5 N1s XPS analysis of the Ru@NDCs-800.

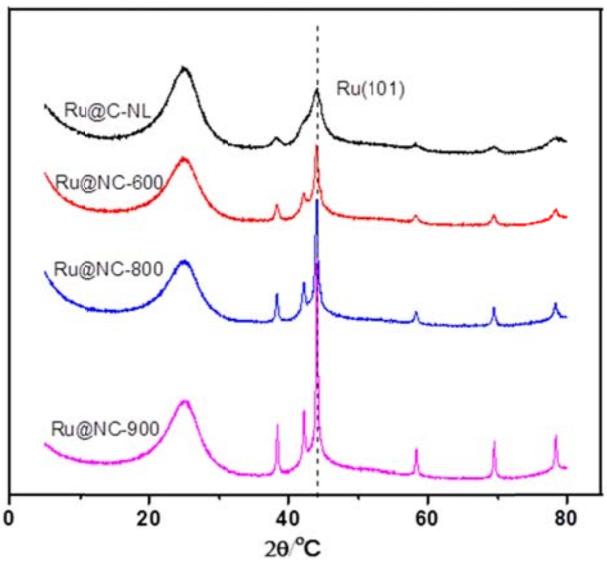

Supplementary Figure 6 XRD analysis of the prepared catalysts.

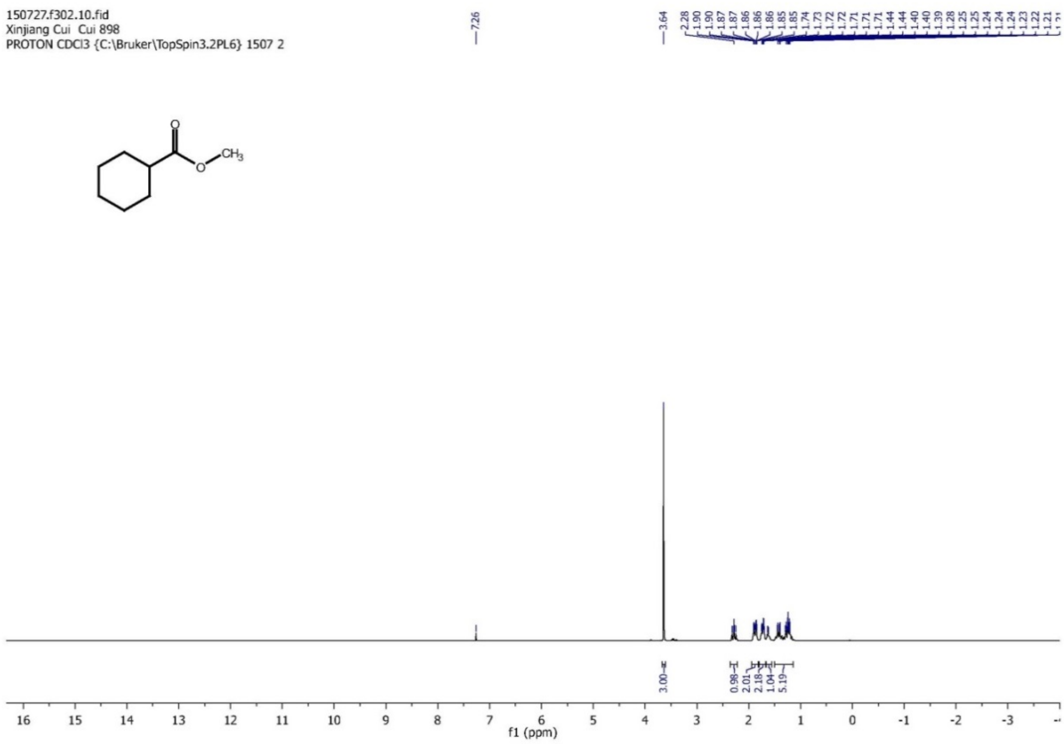

Supplementary Figure 7. ^{1}H NMR (Table 2, entry 1)

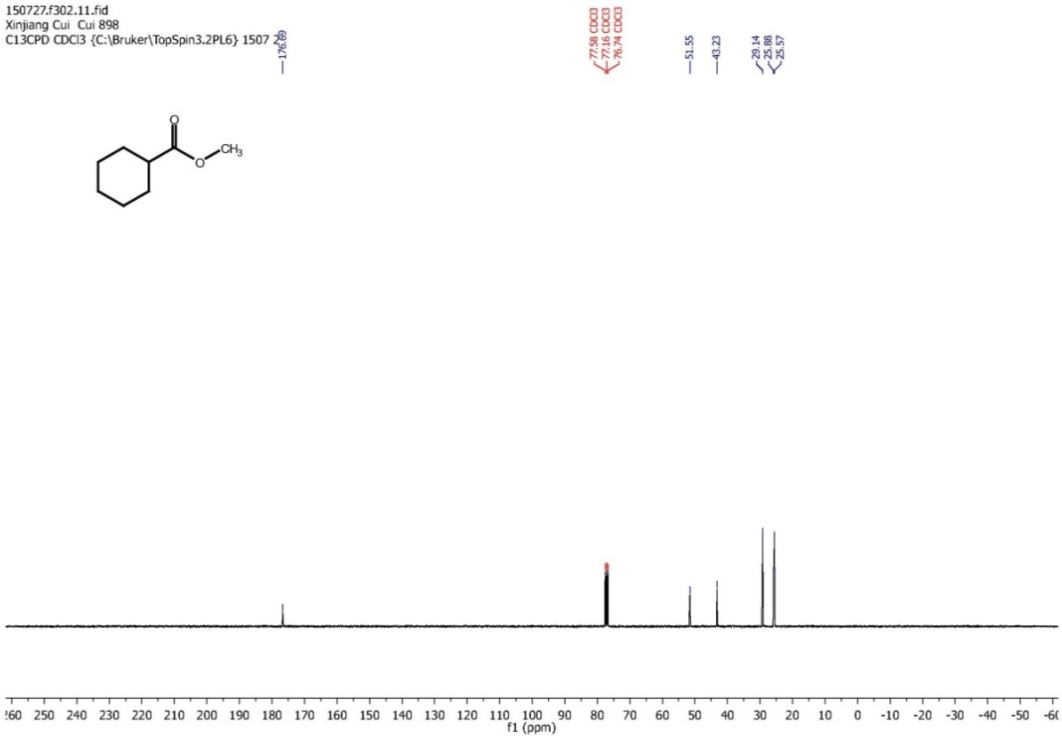

Supplementary Figure 8. ^{13}C NMR (Table 2, entry 1)

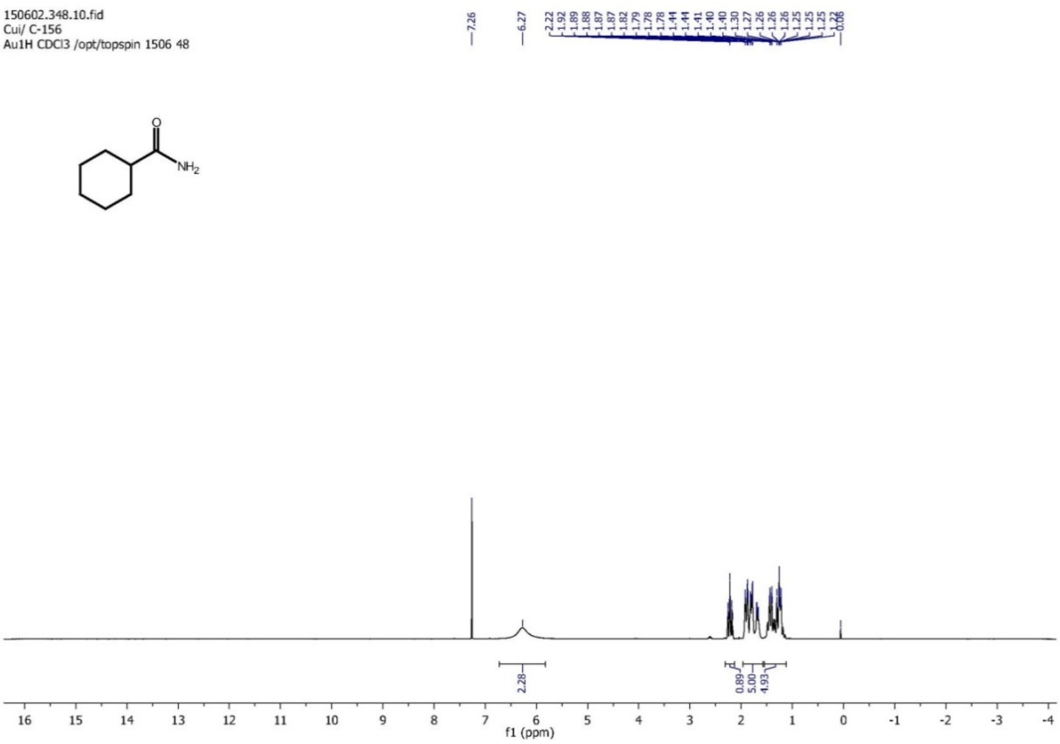

Supplementary Figure 9. ^{1}H NMR (Table 2, entry 2)

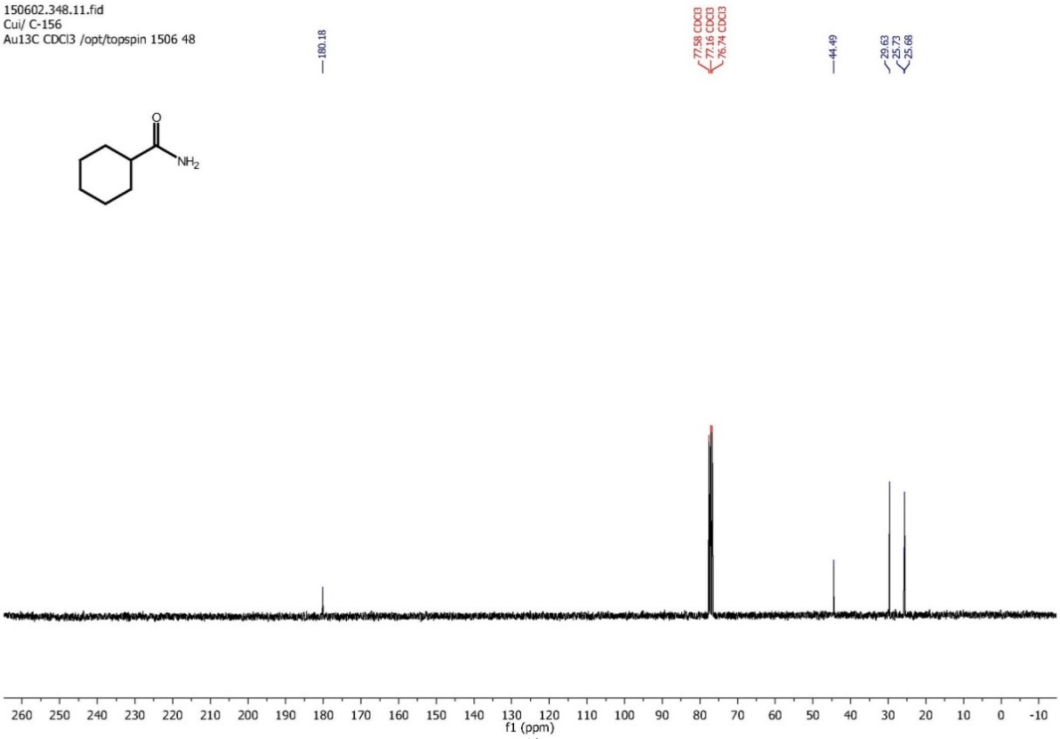

Supplementary Figure 10. ^{13}C NMR (Table 2, entry 2)

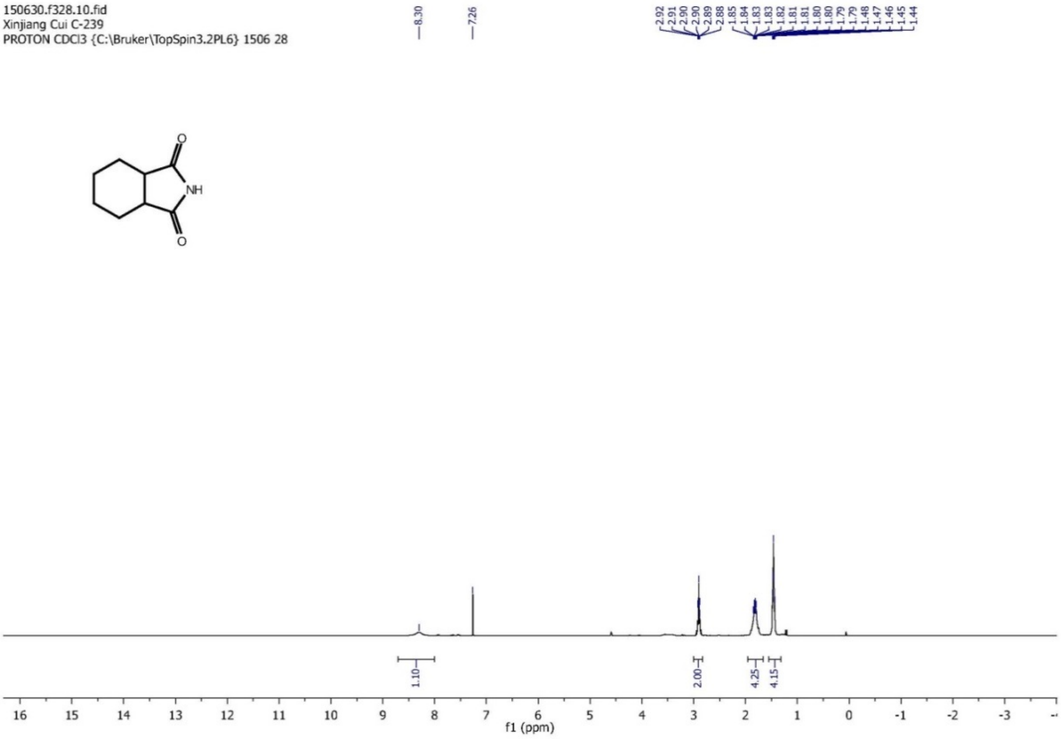

Supplementary Figure 11. ^{1}H NMR (Table 2, entry 3)

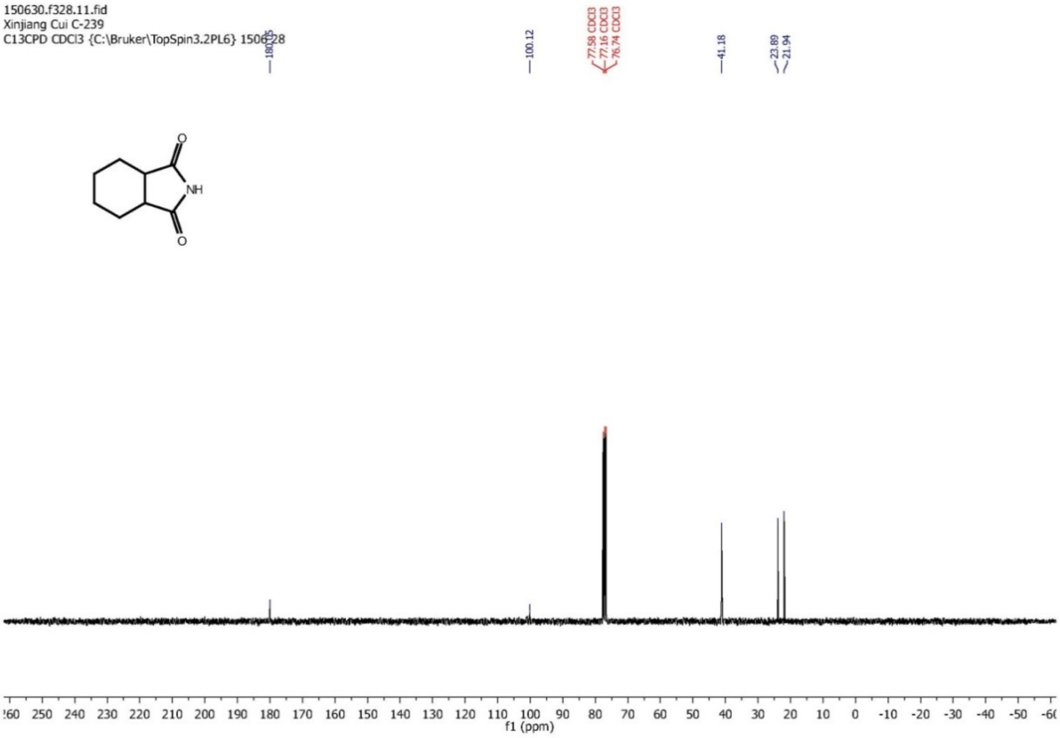

Supplementary Figure 12. ^{13}C NMR (Table 2, entry 3)

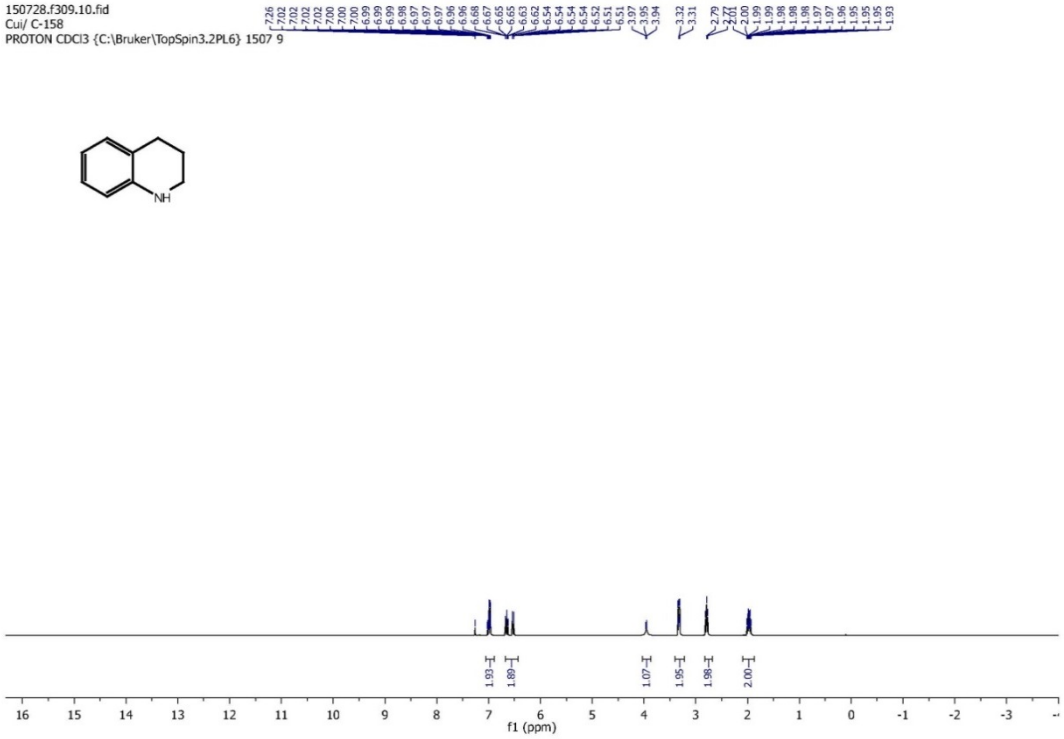

Supplementary Figure 13. ^{1}H NMR (Table 2, entry 4)

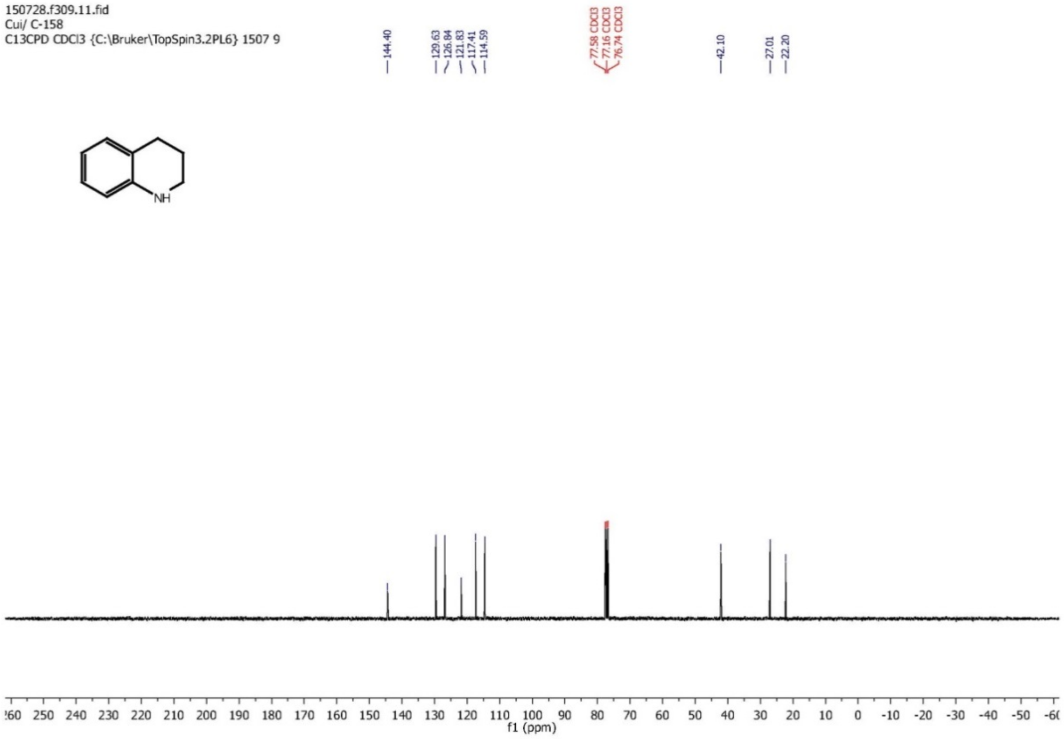

Supplementary Figure 14. ^{13}C NMR (Table 2, entry 4)

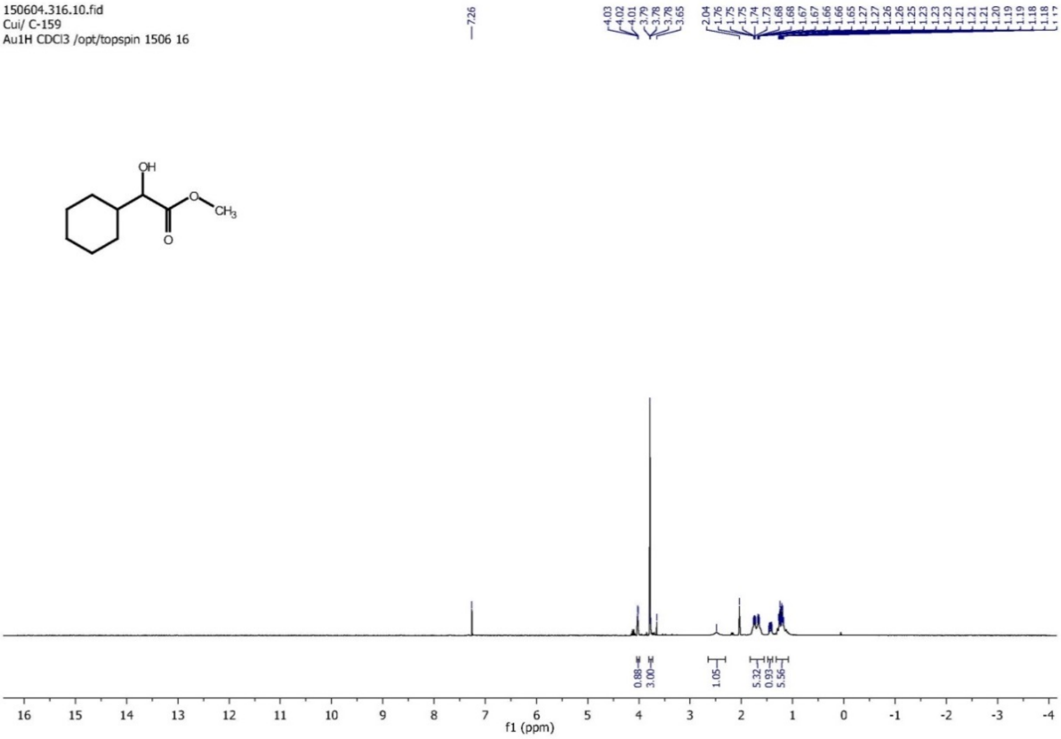

Supplementary Figure 15. ^{1}H NMR (Table 2, entry 5)

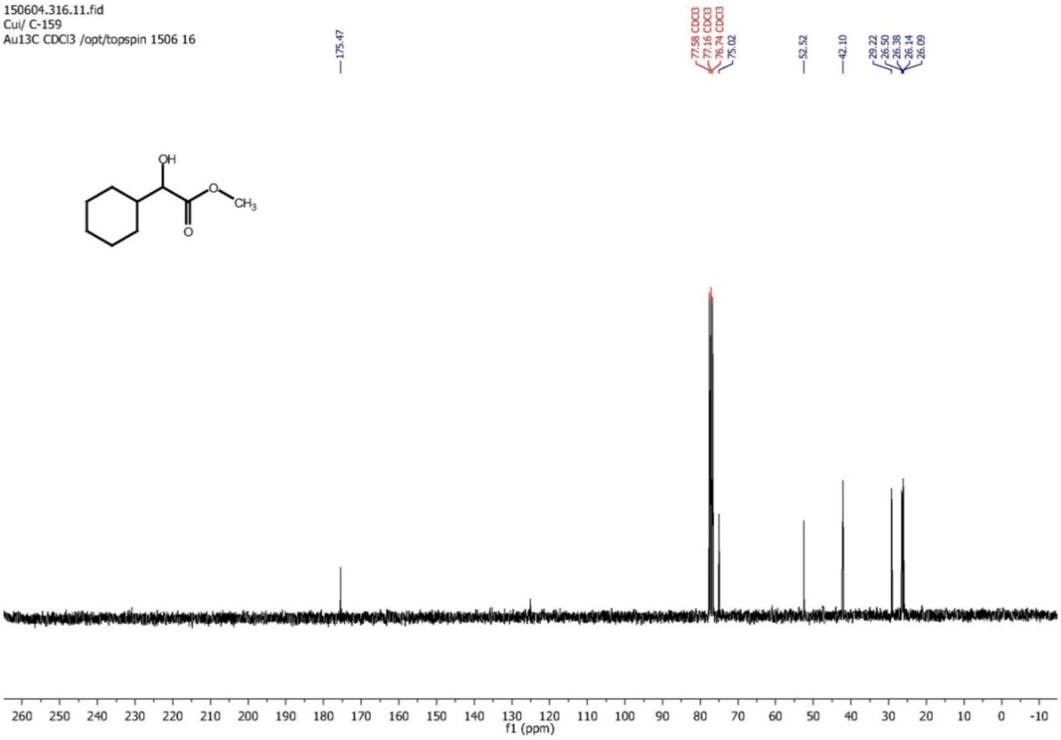

Supplementary Figure 16. ^{13}C NMR (Table 2, entry 5)

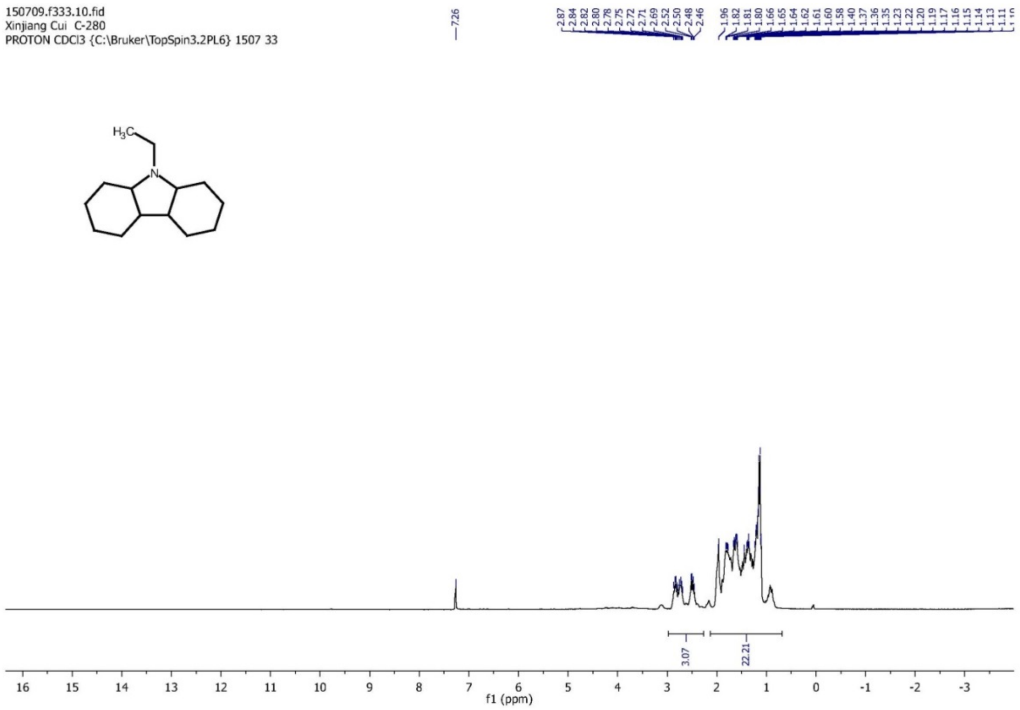

Supplementary Figure 17. ^{1}H NMR (Table 2, entry 8)

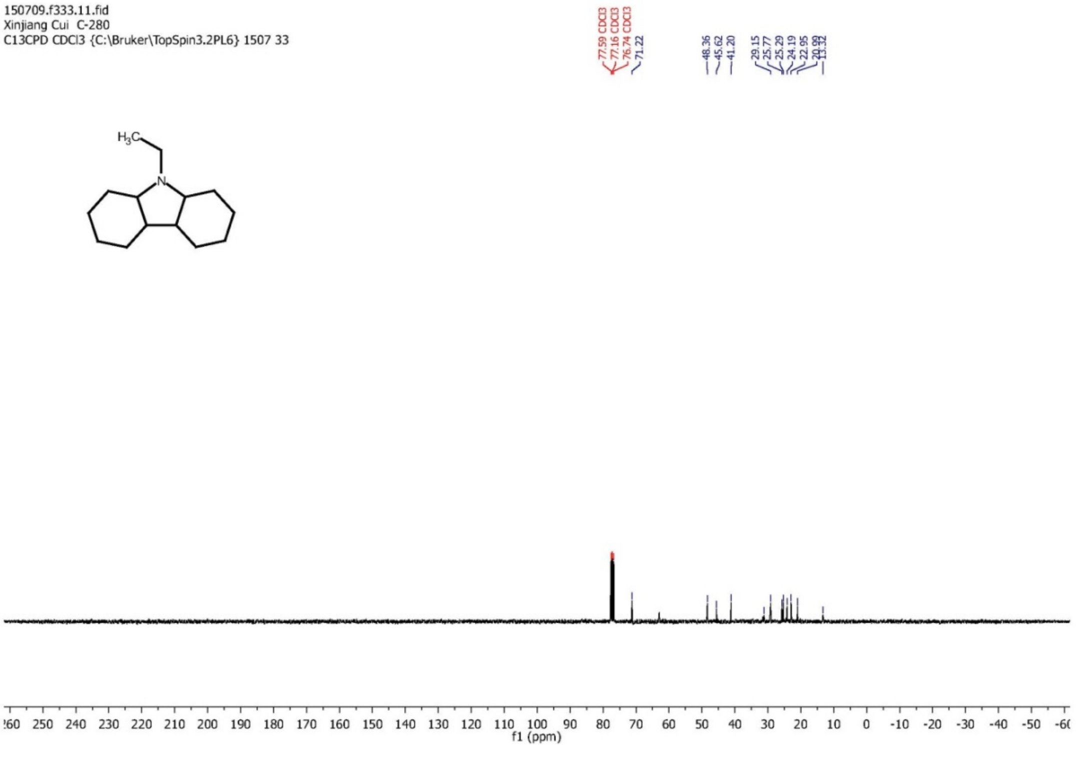

Supplementary Figure 18. ^{13}C NMR (Table 2, entry 8)

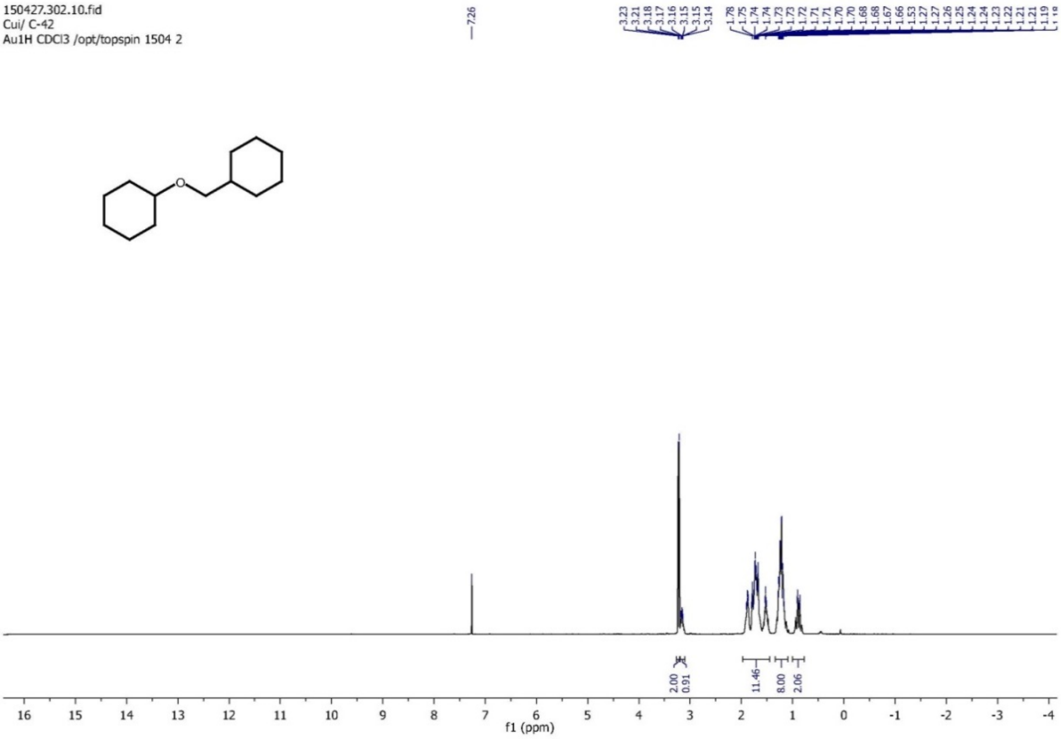

Supplementary Figure 19. ^{1}H NMR of **1a**

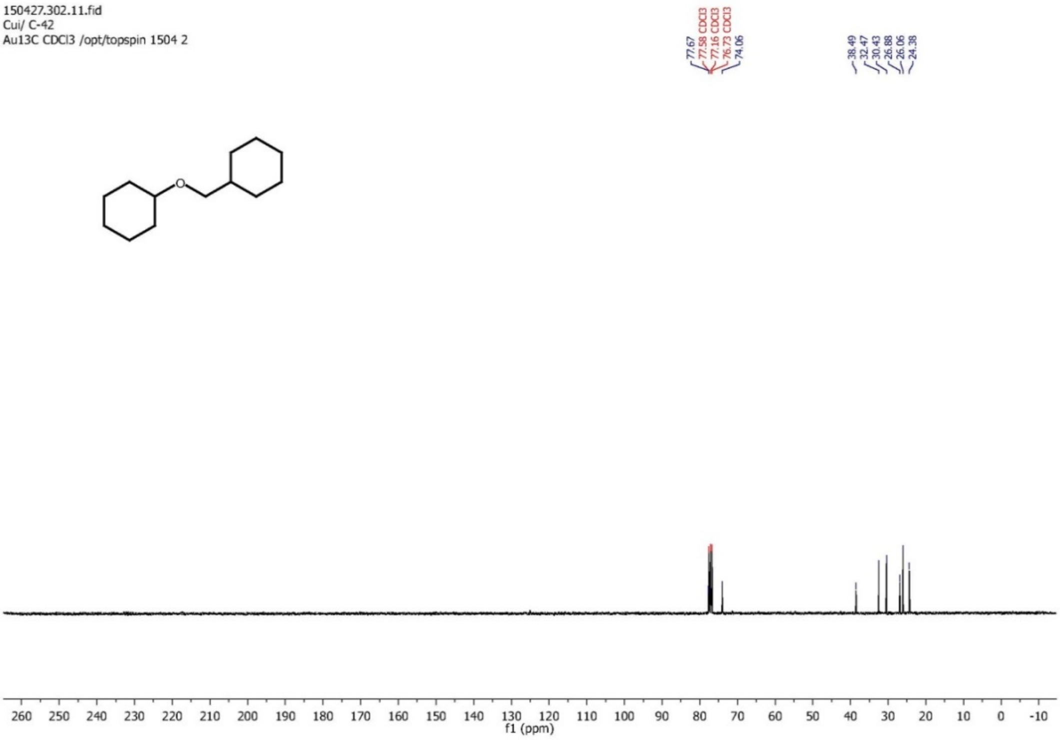

Supplementary Figure 20. ^{13}C NMR of **1a**

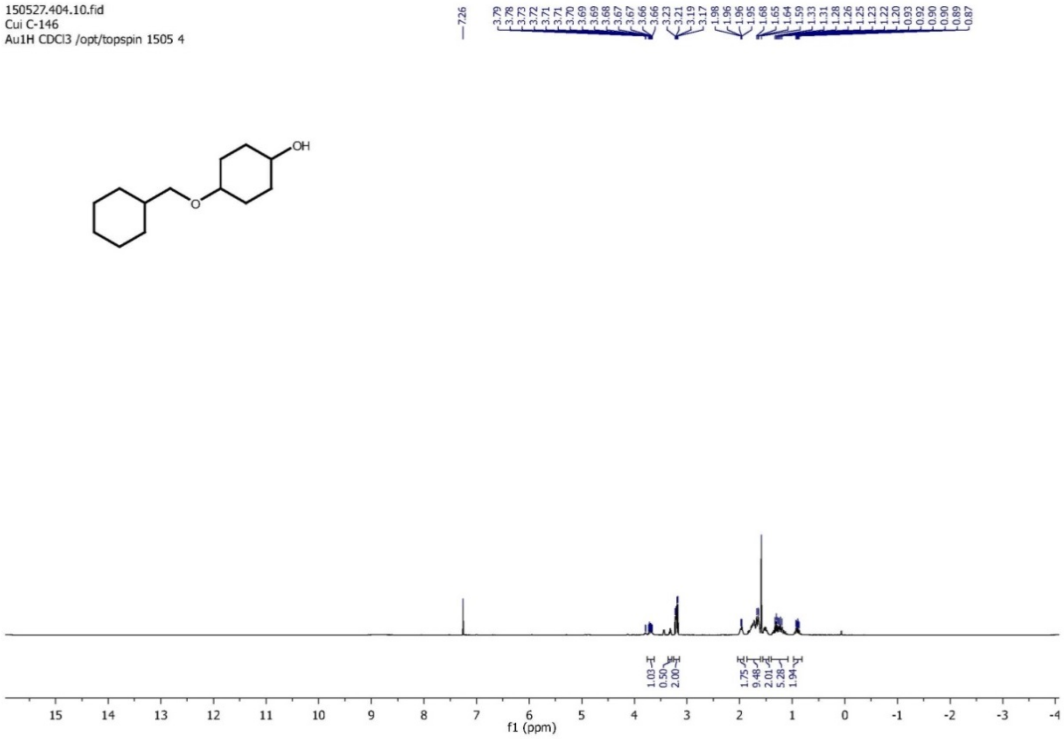

Supplementary Figure 21. ^{1}H NMR of **1b**

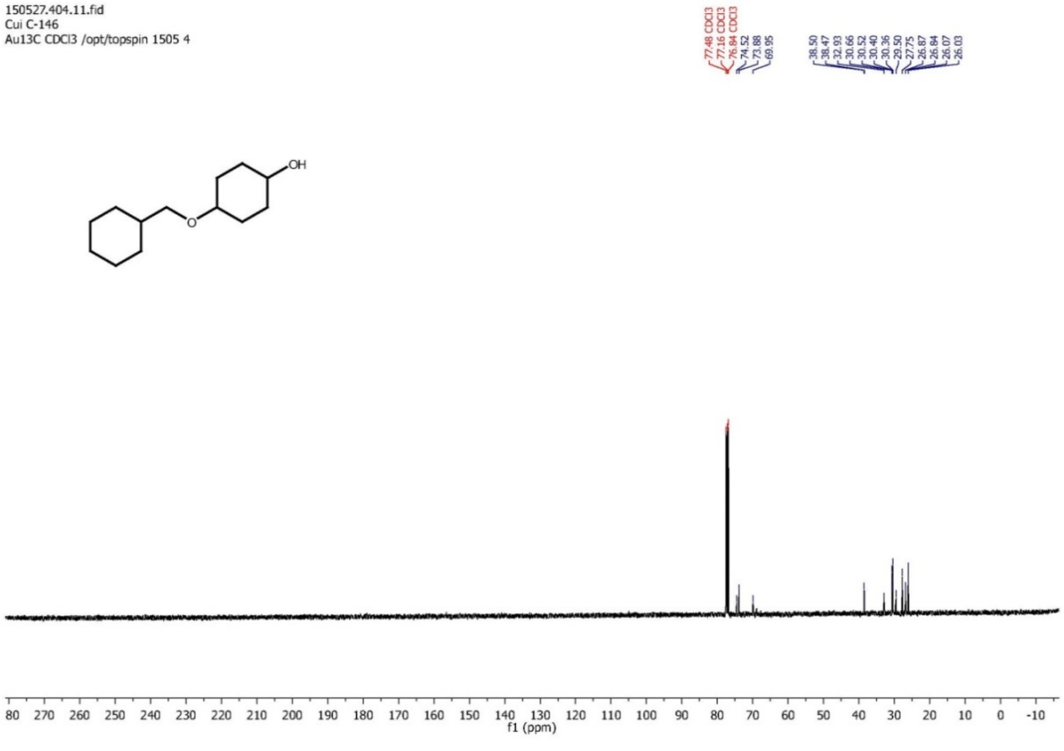

Supplementary Figure 22. ^{1}H NMR of **1b**

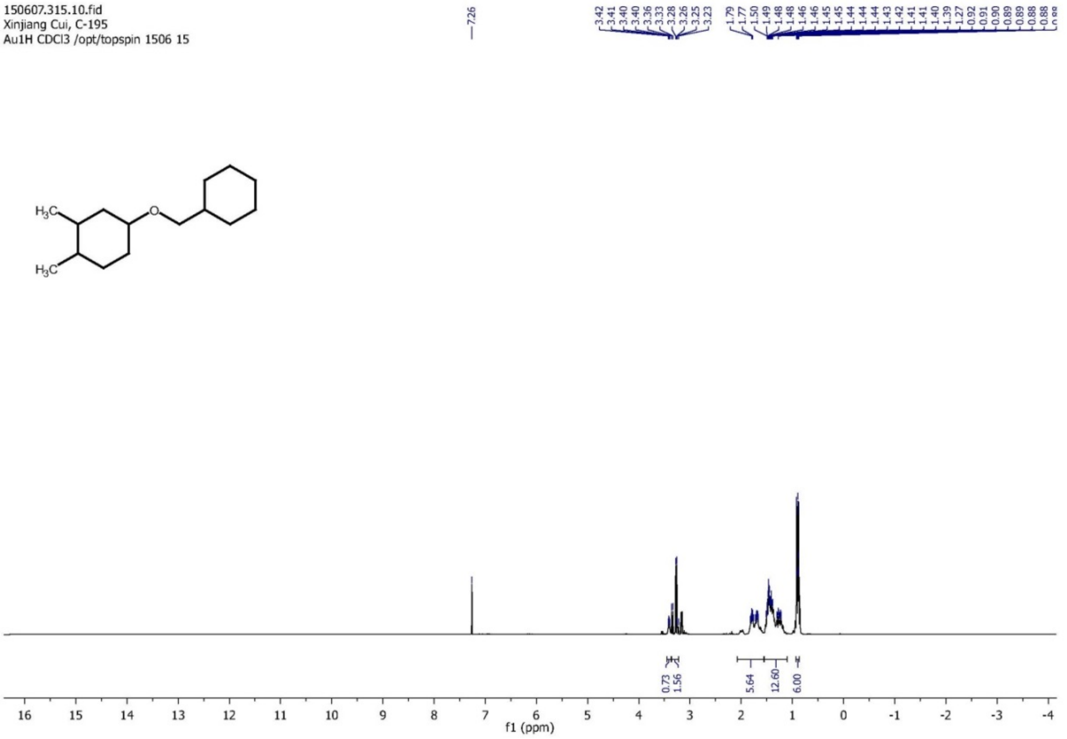

Supplementary Figure 23. ^{1}H NMR of **1c**

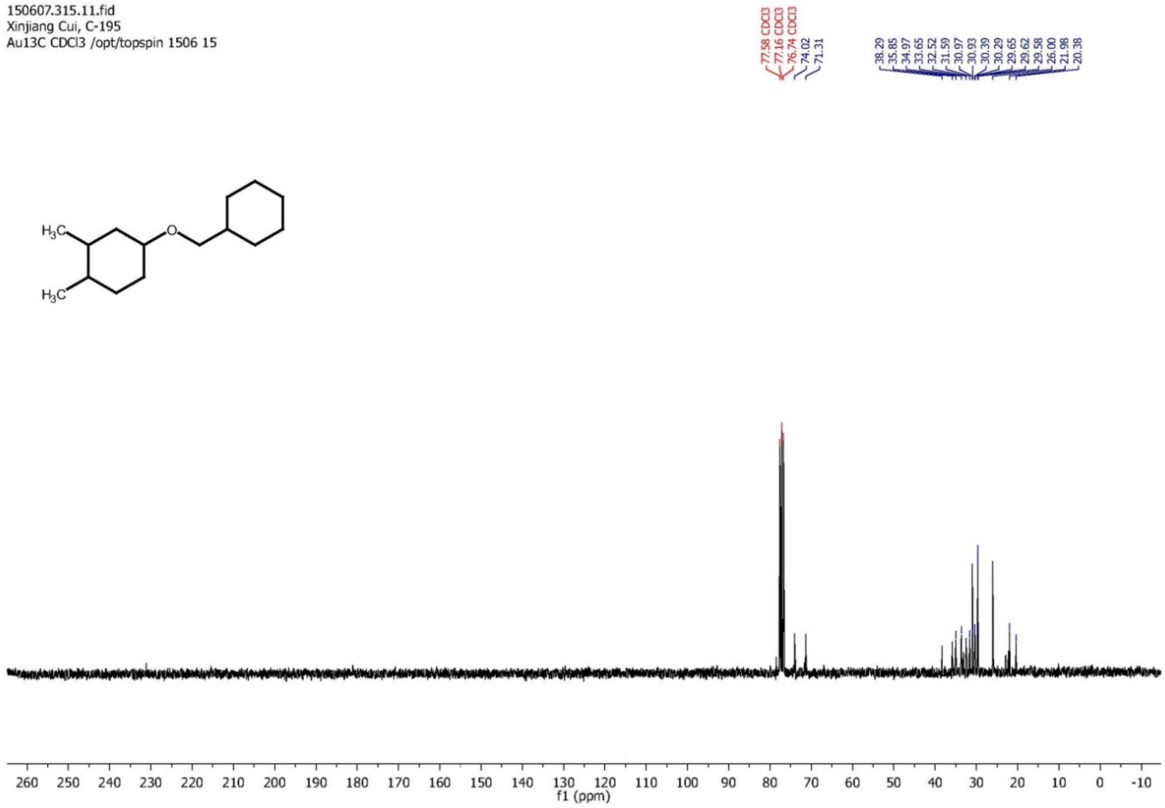

Supplementary Figure 24. ^{1}H NMR of **1c**

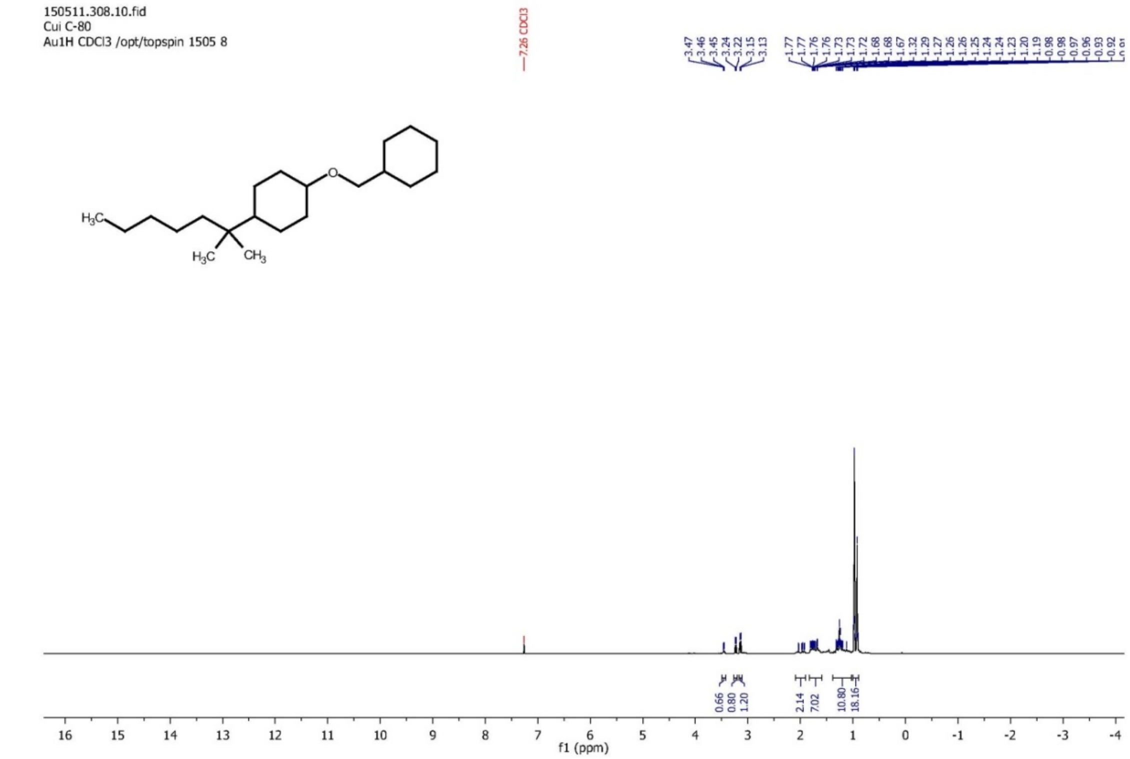

Supplementary Figure 25. ^{1}H NMR of **1d**

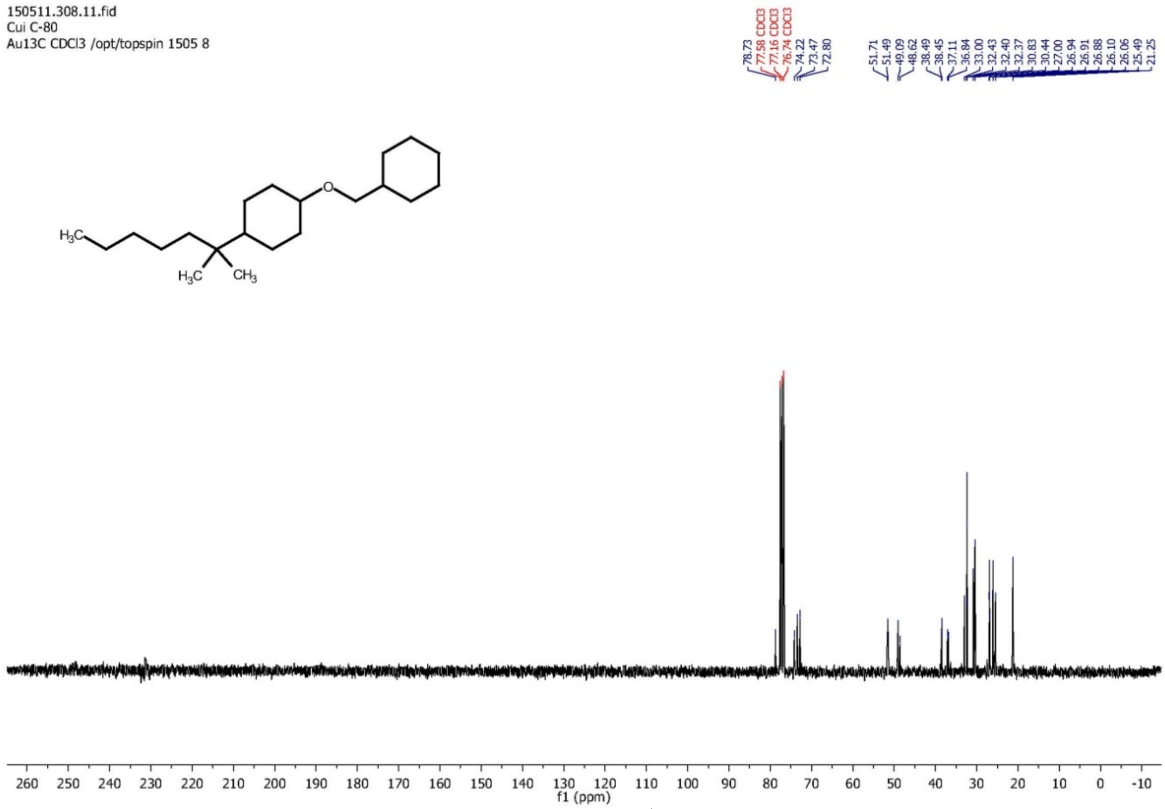

Supplementary Figure 26. ^{1}H NMR of **1d**

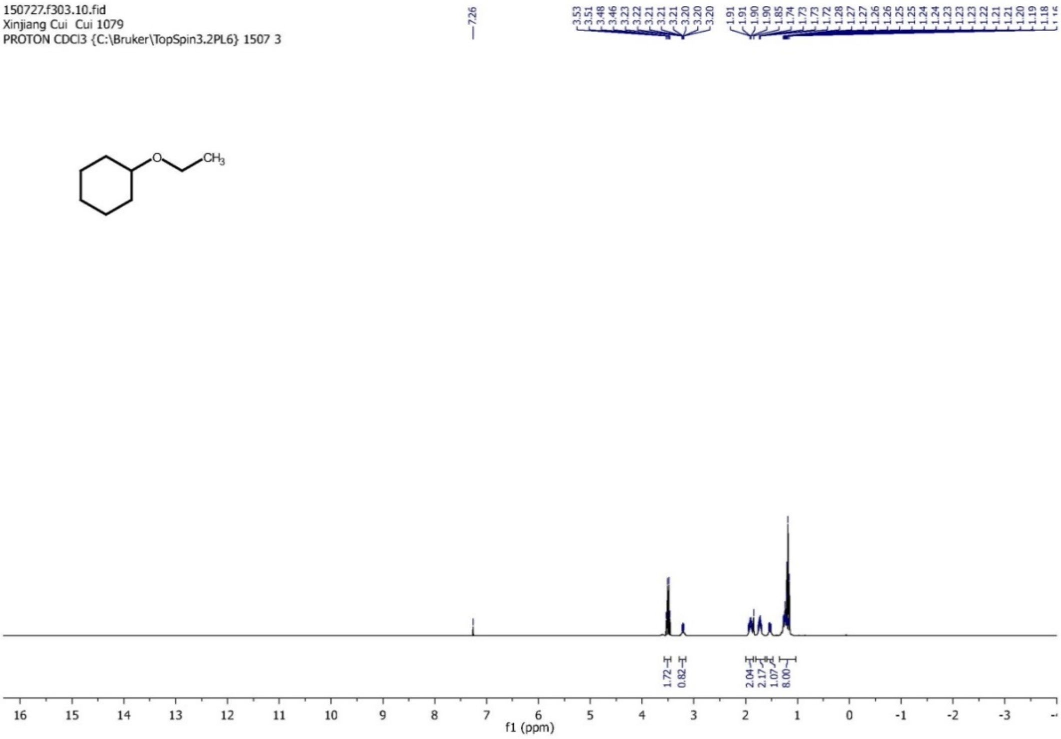

Supplementary Figure 27. ^{1}H NMR (Table 3, entry 2)

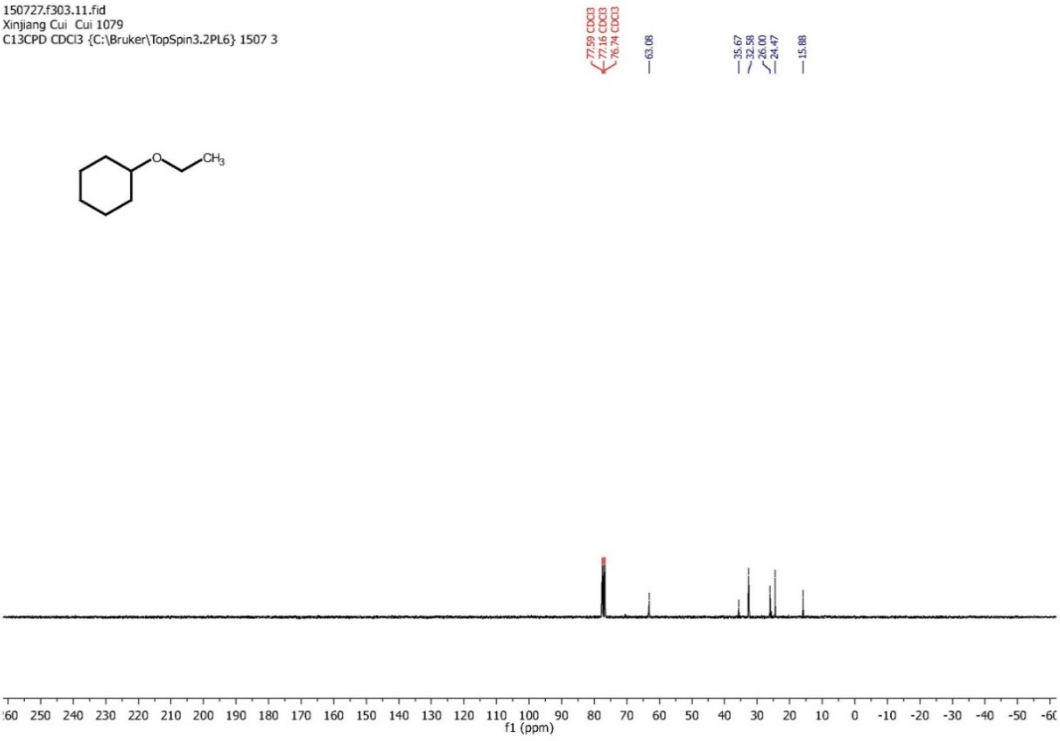

Supplementary Figure 28. ^{13}C NMR (Table 3, entry 2)

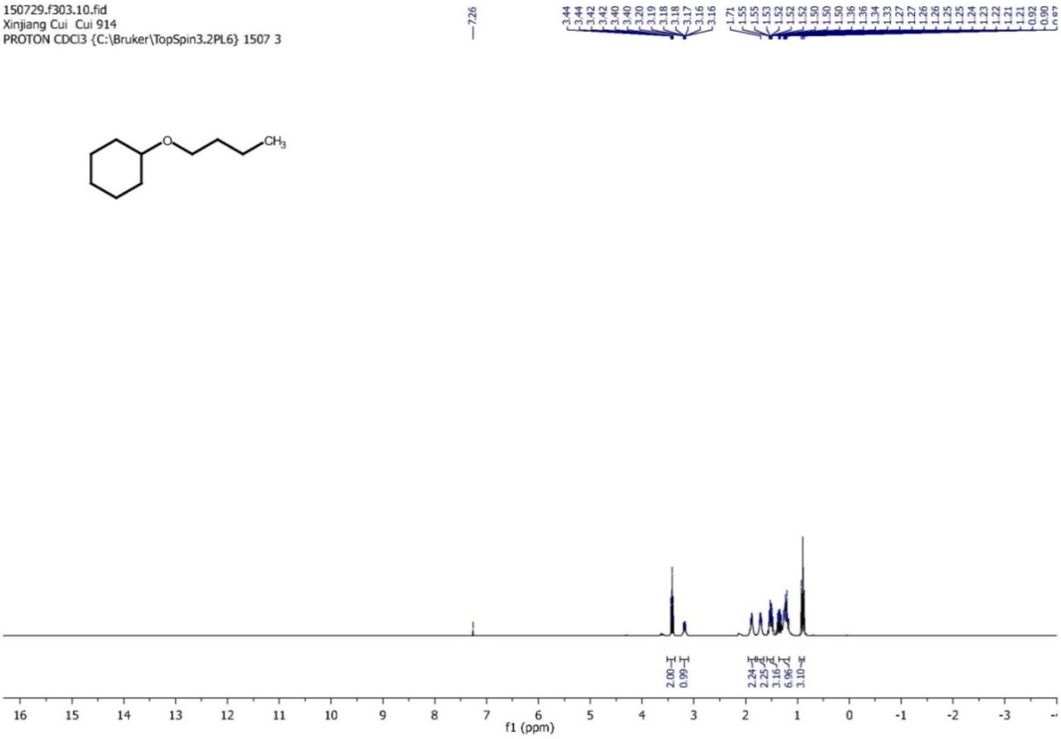

Supplementary Figure 29. ^{1}H NMR (Table 3, entry 3)

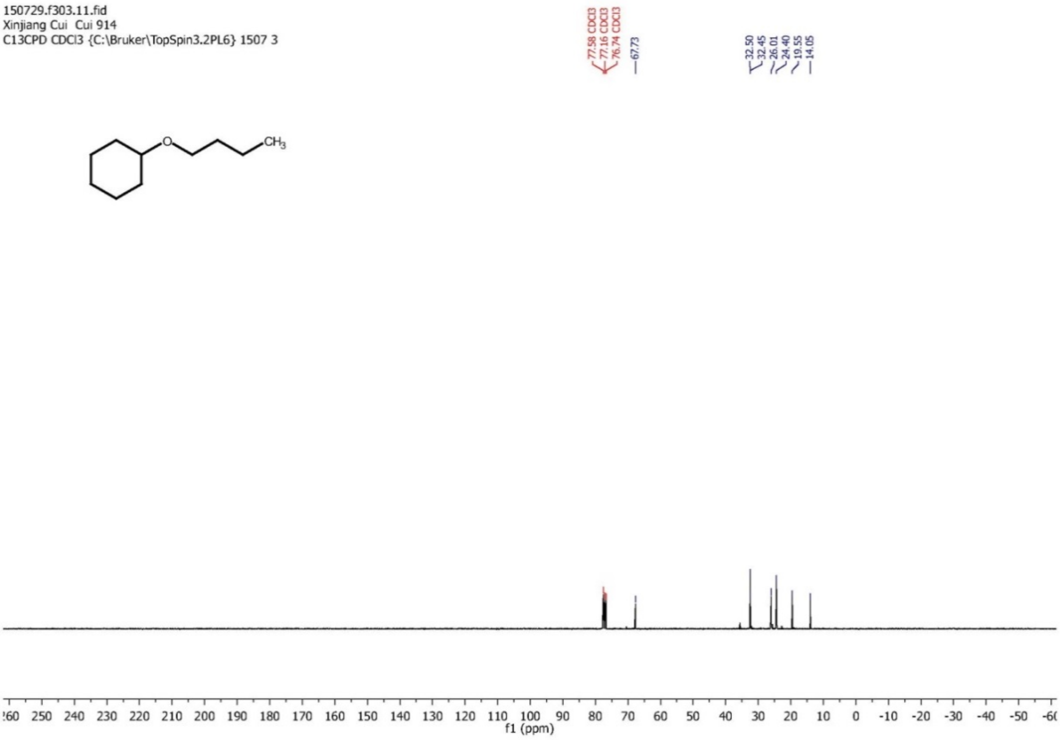

Supplementary Figure 30. ^{13}C NMR (Table 3, entry 3)

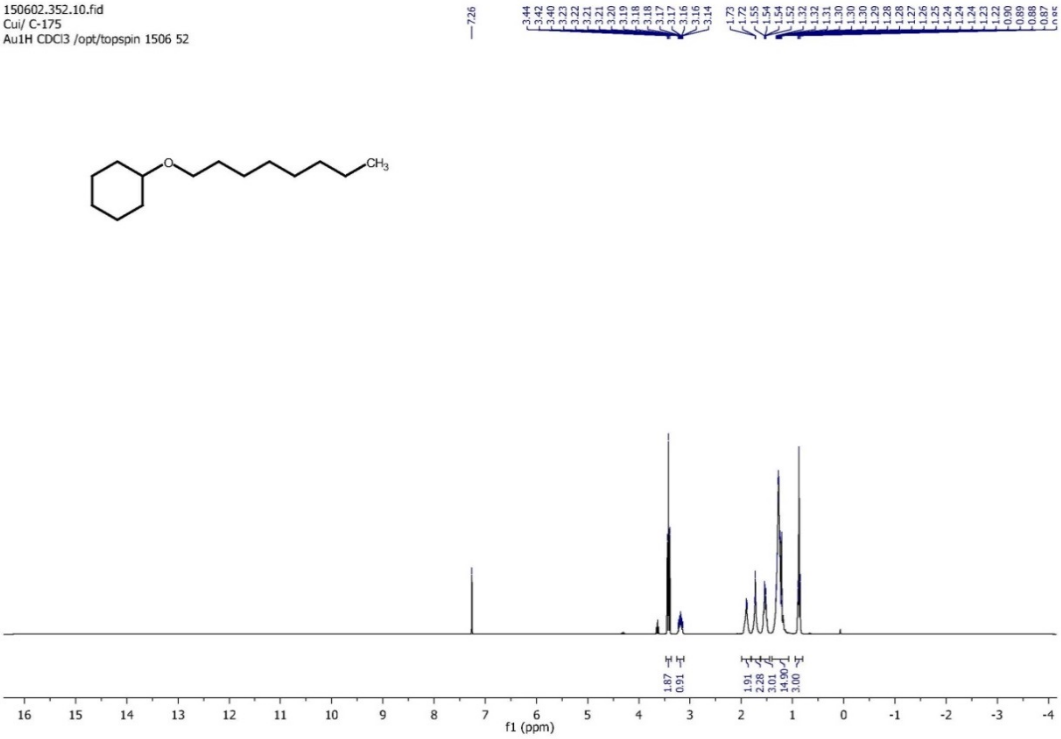

Supplementary Figure 31. ^{1}H NMR (Table 3, entry 4)

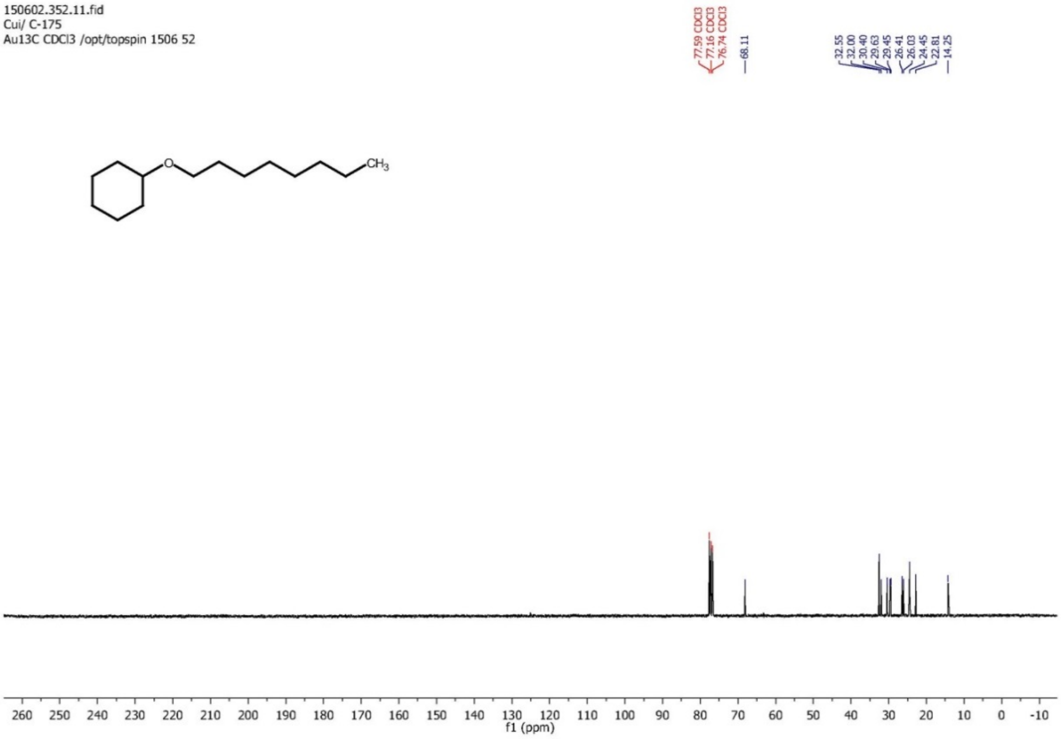

Supplementary Figure 32. ^{13}C NMR (Table 3, entry 4)

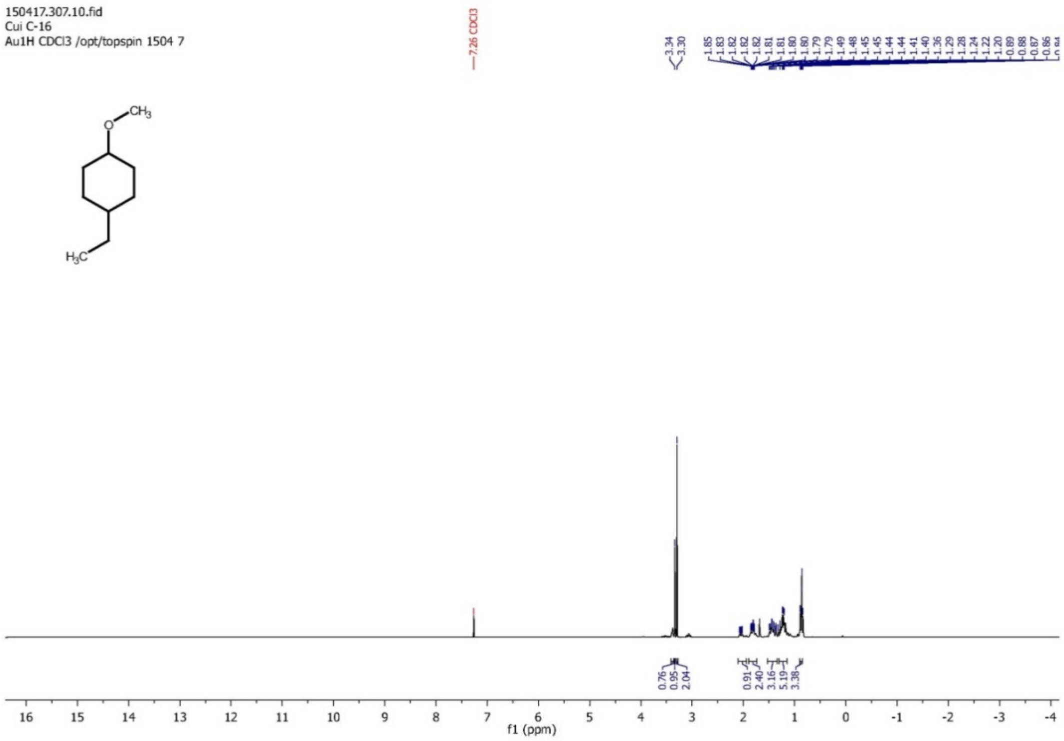

Supplementary Figure 33. ^{1}H NMR (Table 3, entry 5)

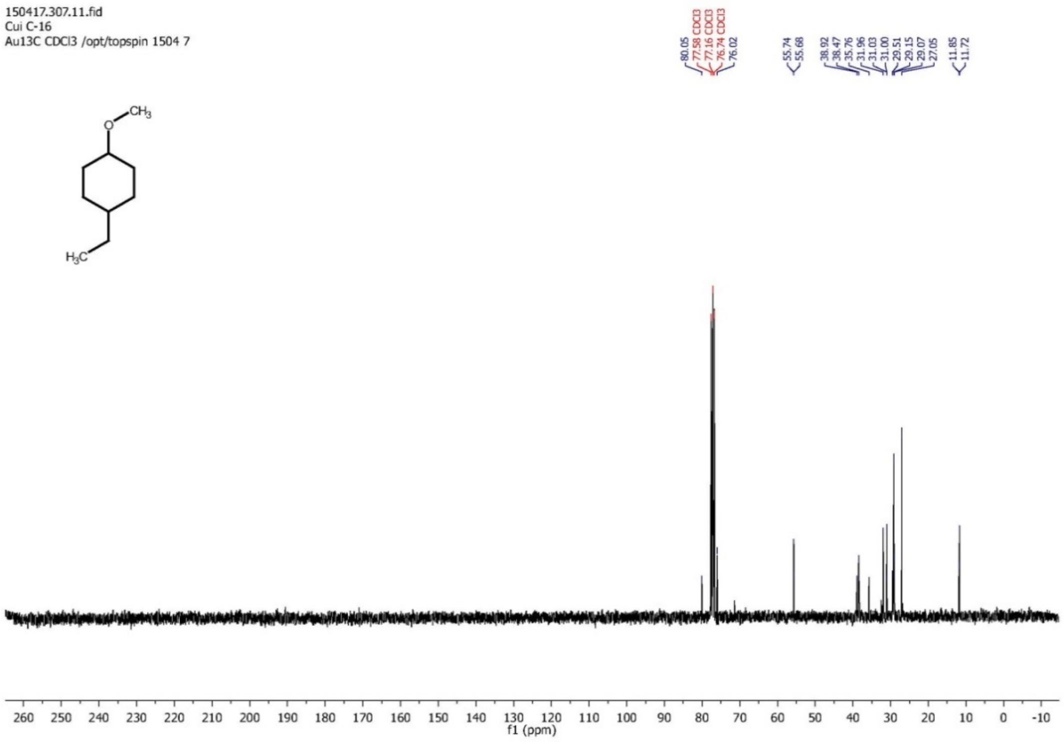

Supplementary Figure 34. ^{13}C NMR (Table 3, entry 5)

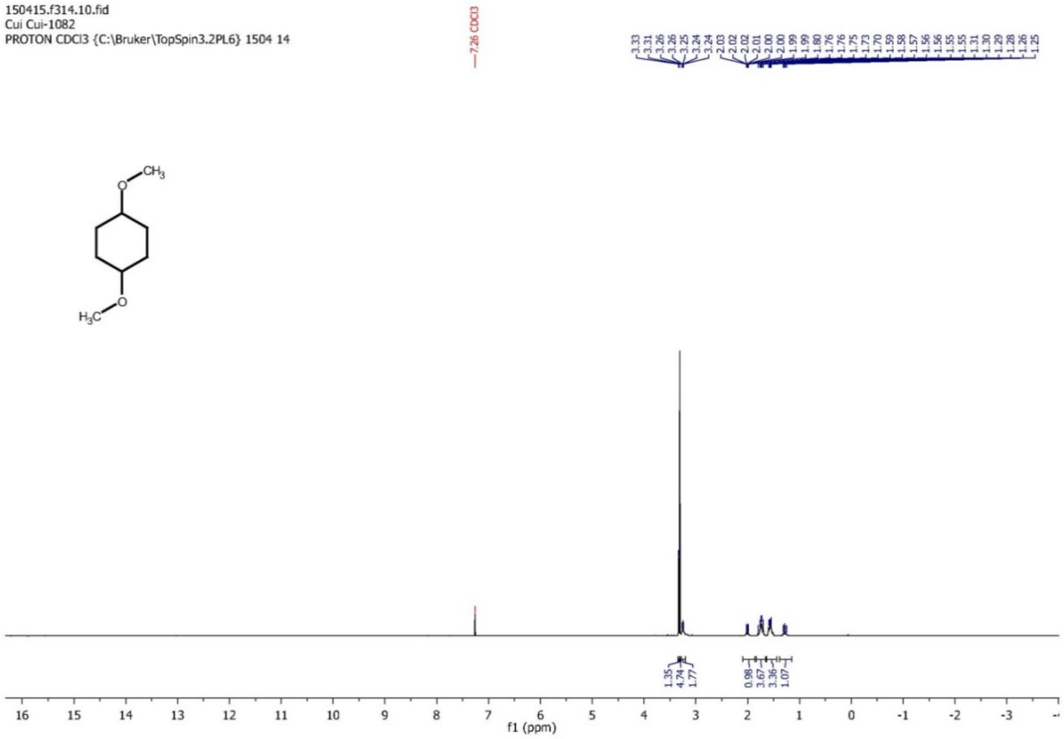

Supplementary Figure 35. ^{1}H NMR (Table 3, entry 6)

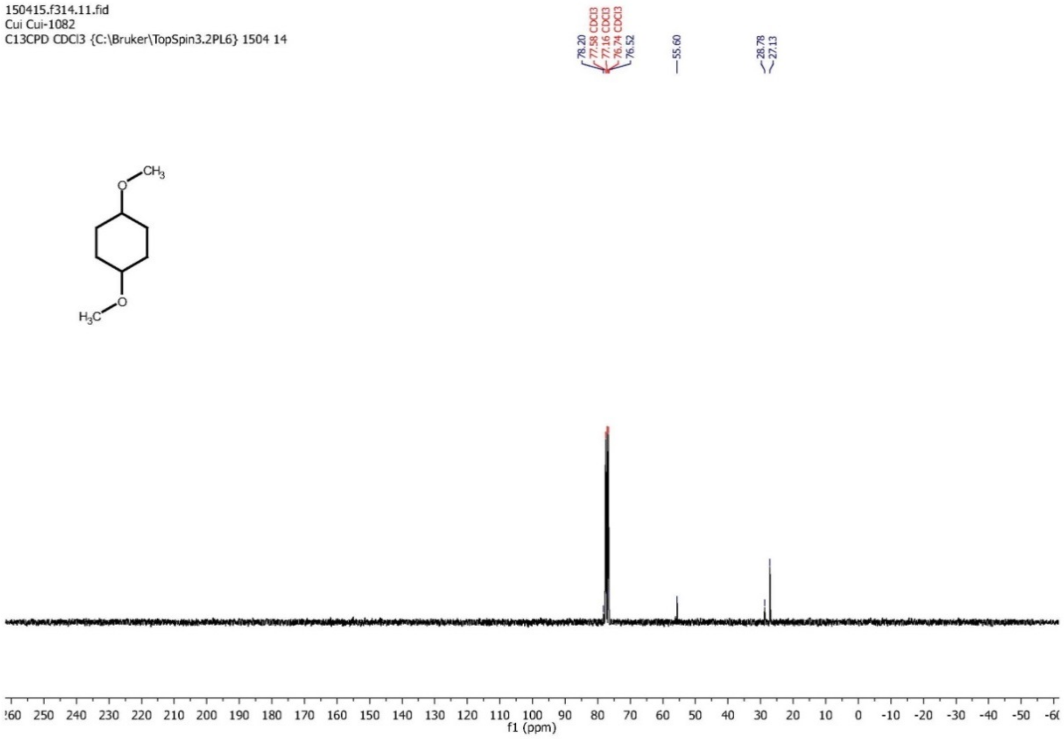

Supplementary Figure 36. ^{13}C NMR (Table 3, entry 6)

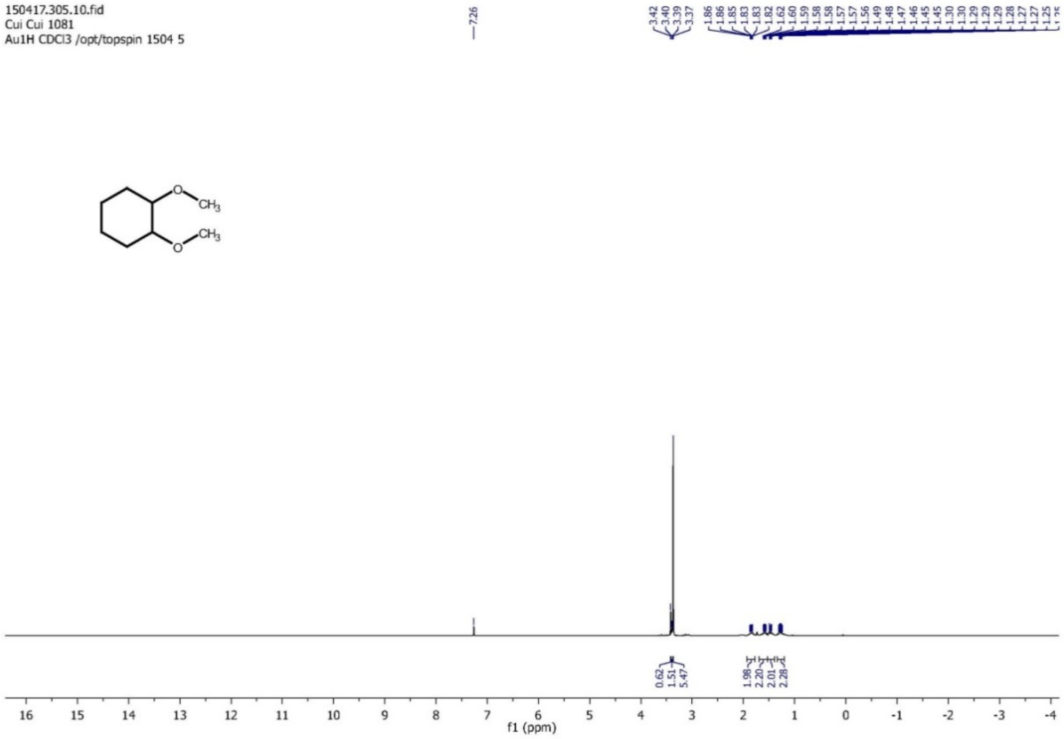

Supplementary Figure 37. ^{1}H NMR (Table 3, entry 7)

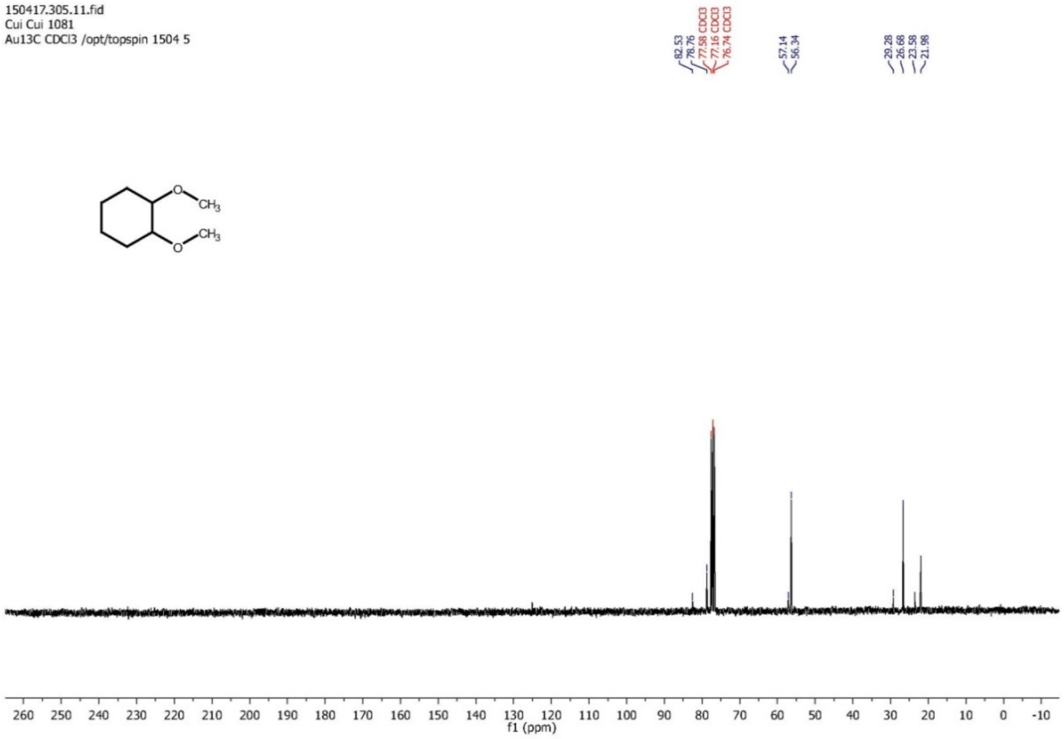

Supplementary Figure 38. ^{13}C NMR (Table 3, entry 7)

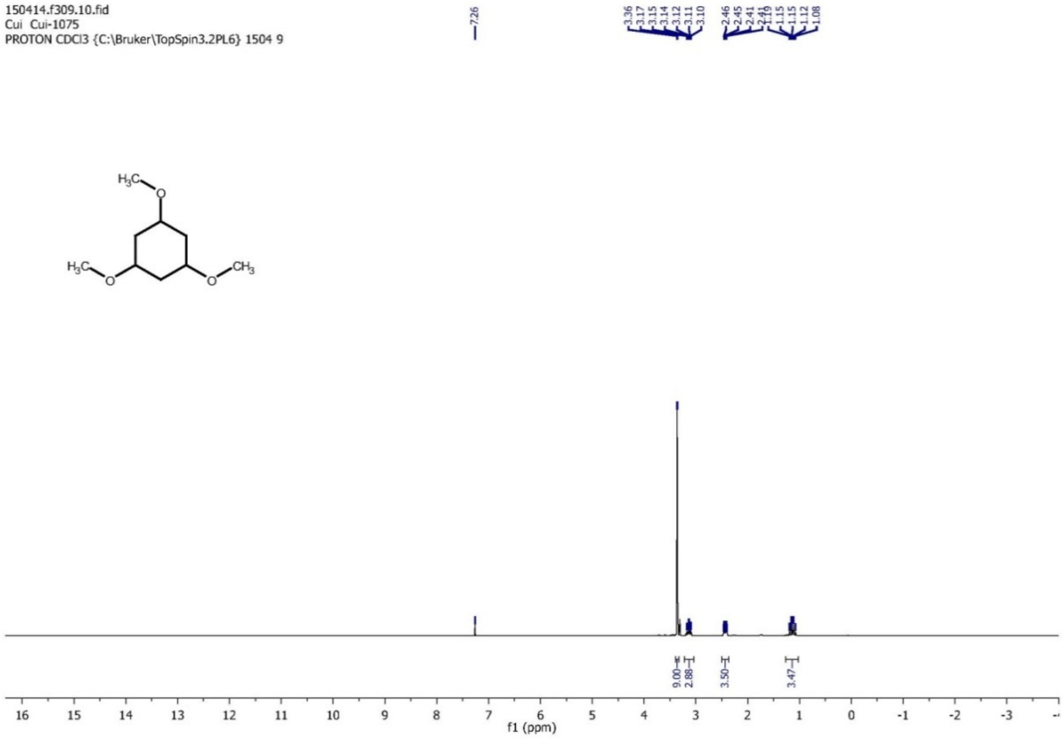

Supplementary Figure 39. ^{1}H NMR (Table 3, entry 8)

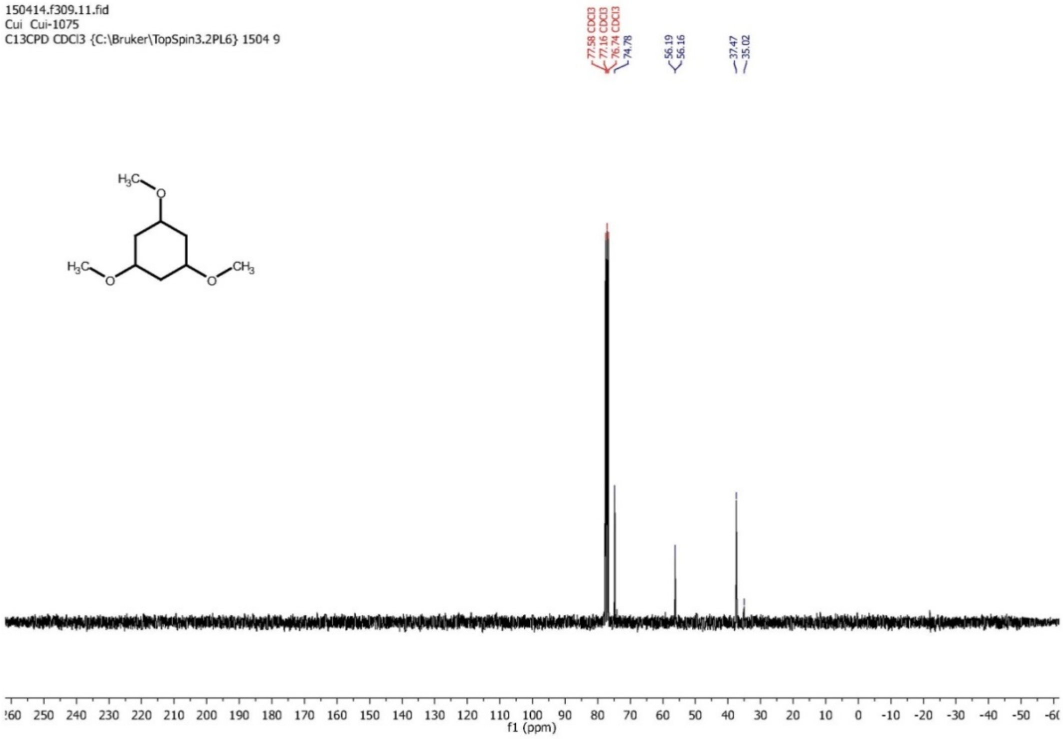

Supplementary Figure 40. ^{13}C NMR (Table 3, entry 8)

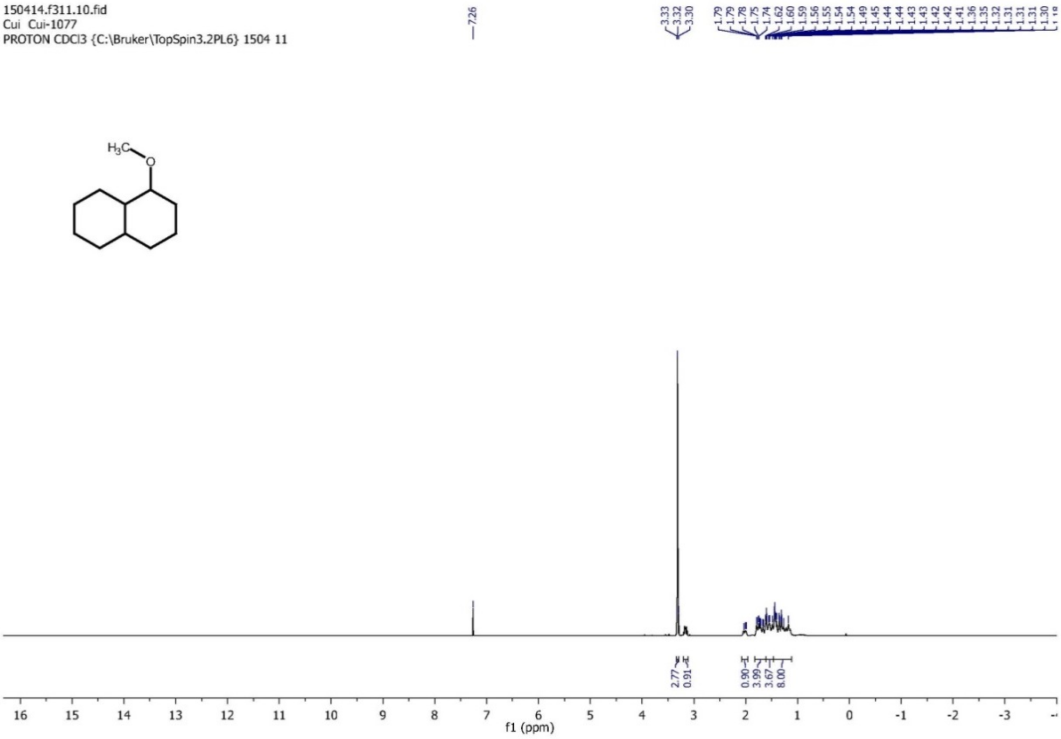

Supplementary Figure 41. ^{1}H NMR (Table 3, entry 9)

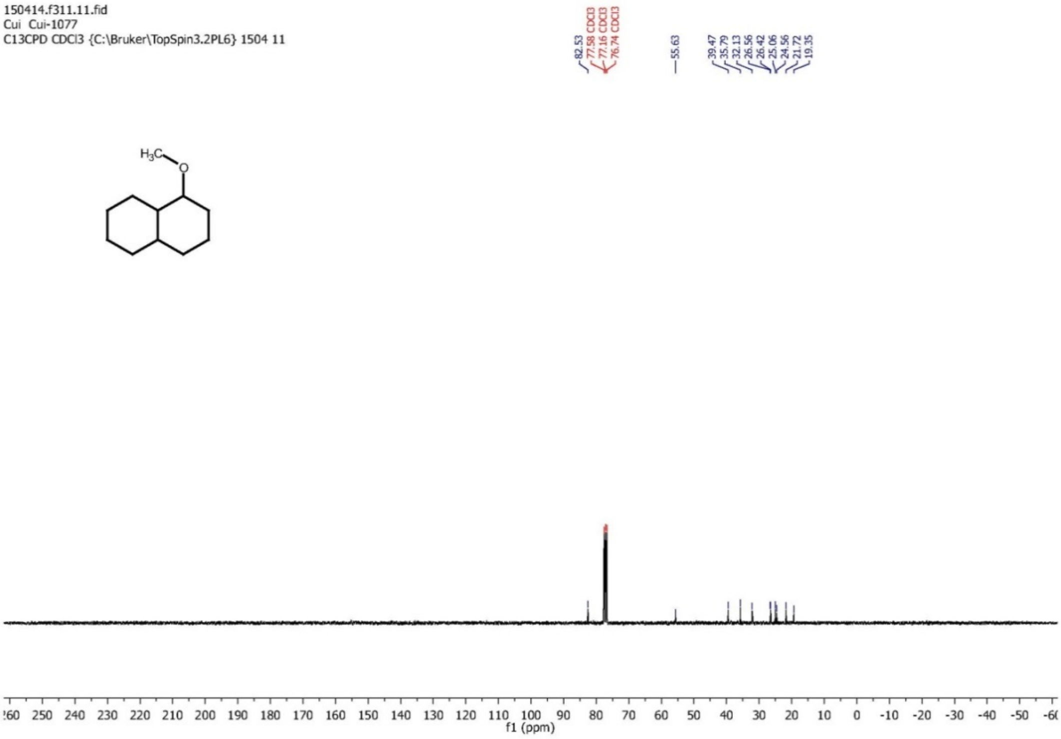

Supplementary Figure 42. ^{13}C NMR (Table 3, entry 9)

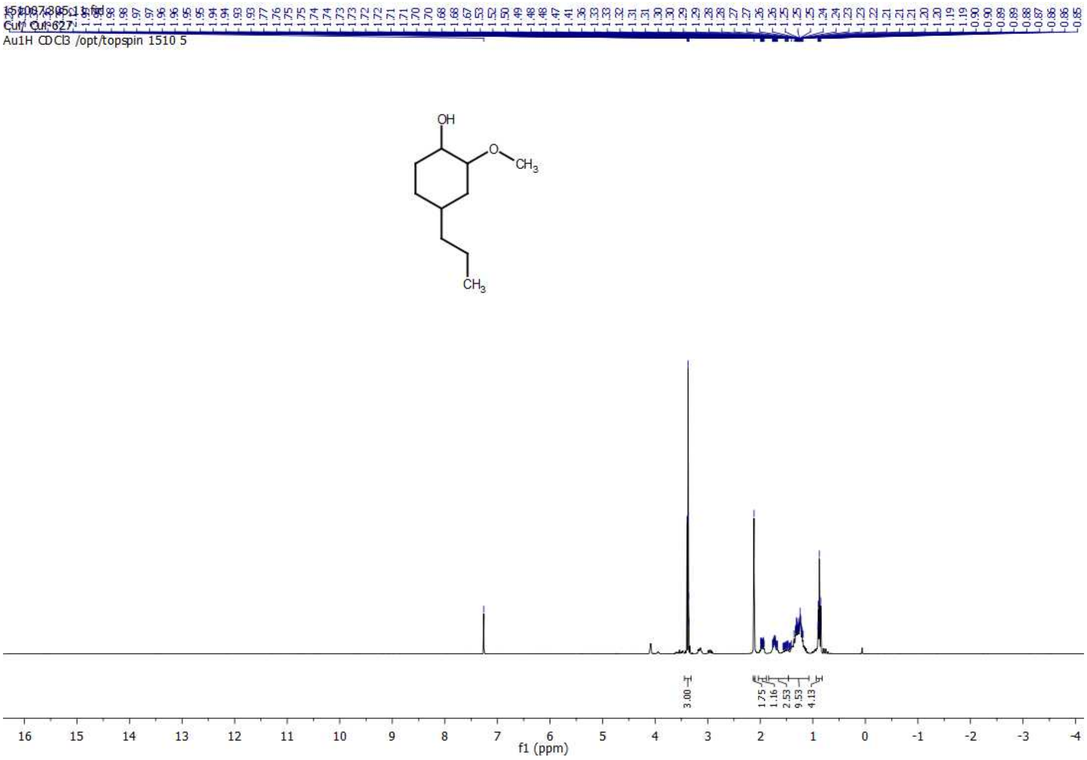

Supplementary Figure 43. ^{1}H NMR (Table 3, entry 13)

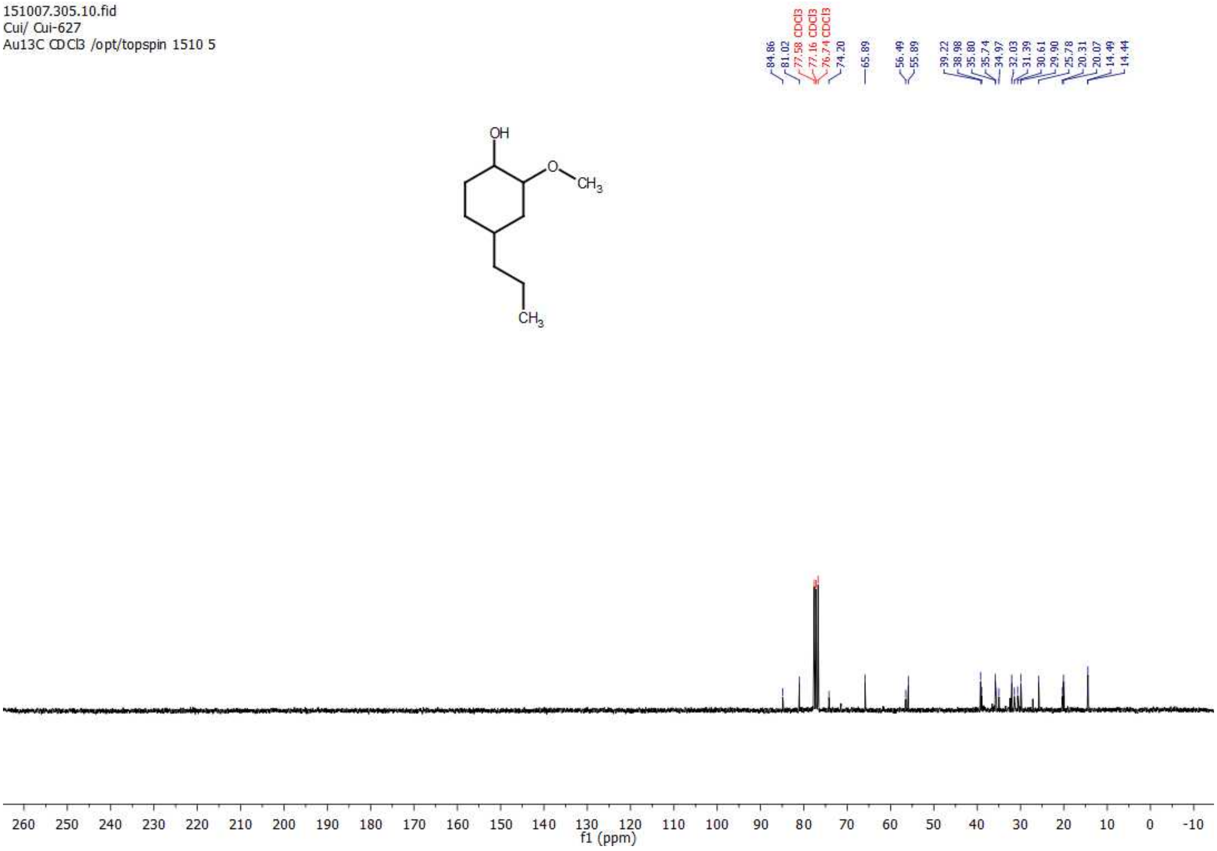

Supplementary Figure 44. ^{13}C NMR (Table 3, entry 13)

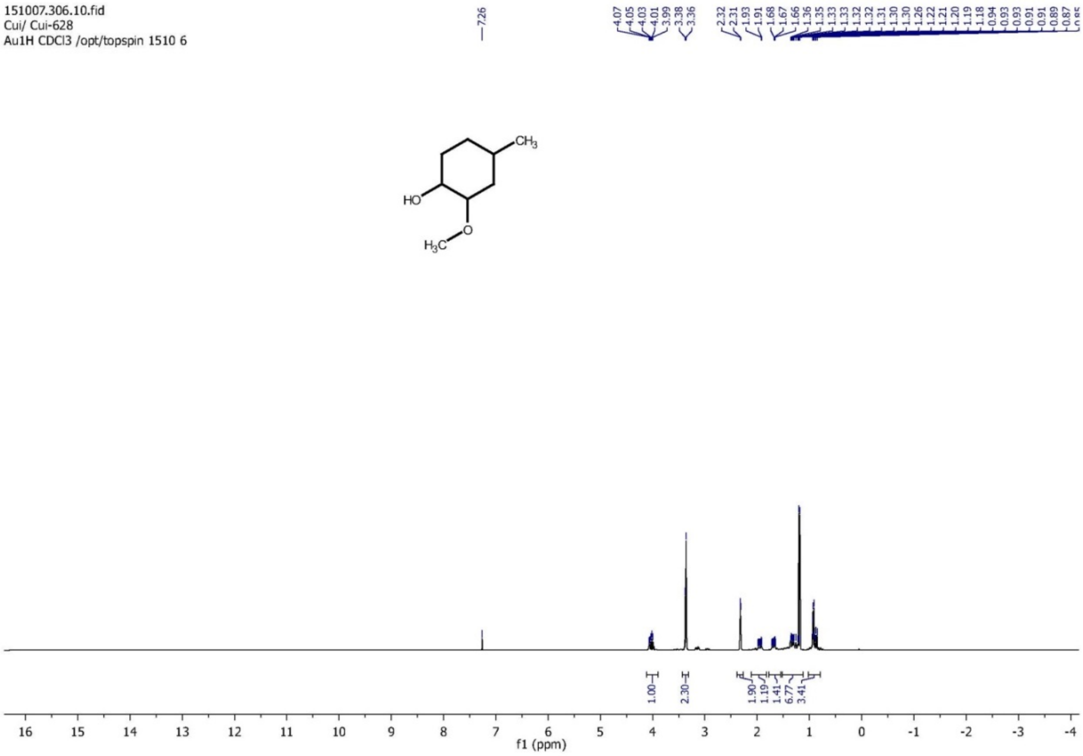

Supplementary Figure 45. ^{1}H NMR (Table 3, entry 14)

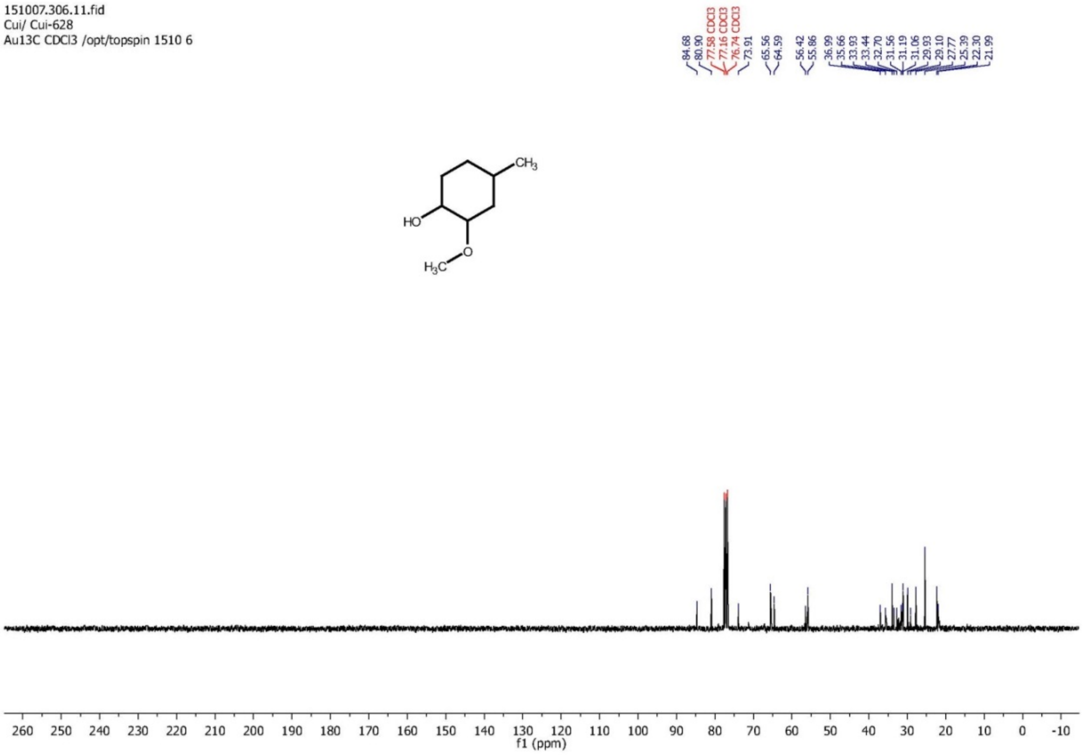

Supplementary Figure 46. ^{13}C NMR (Table 3, entry 14)

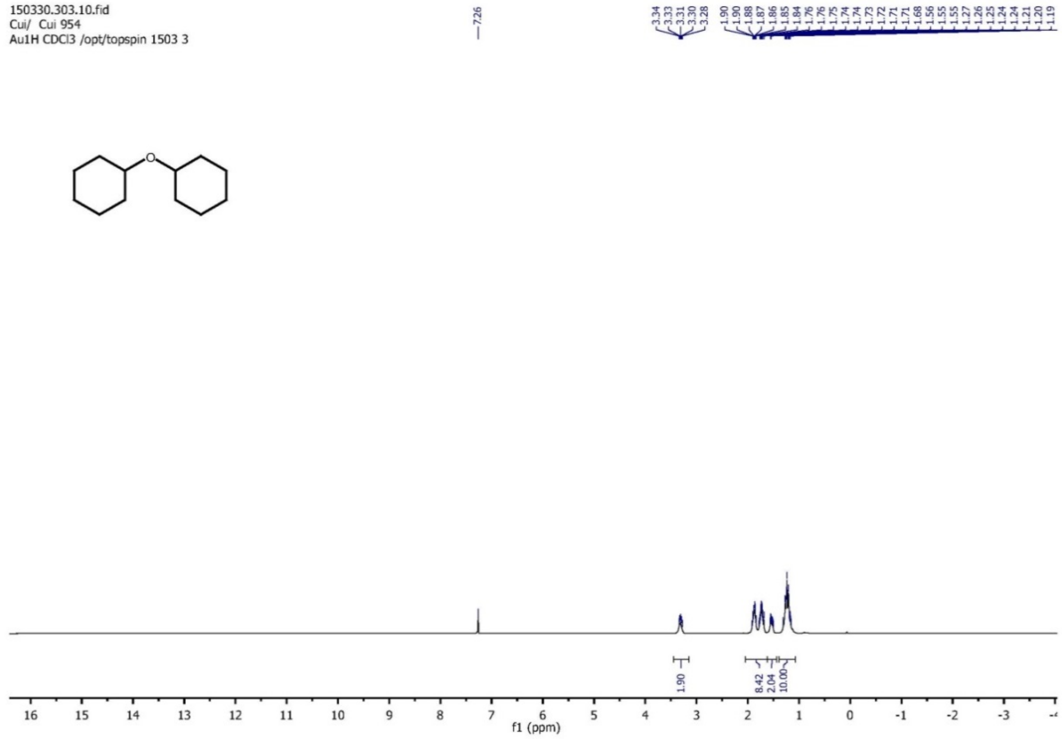

Supplementary Figure 47. ^{1}H NMR (Table 4, entry 1)

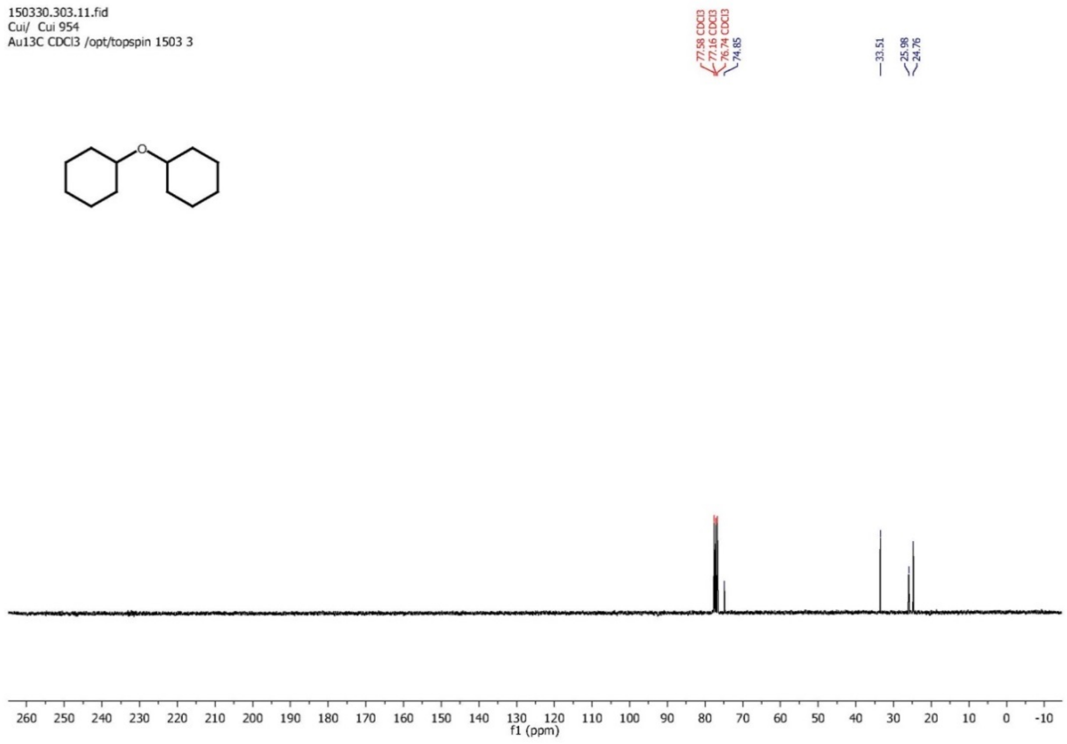

Supplementary Figure 48. ^{13}C NMR (Table 4, entry 1)

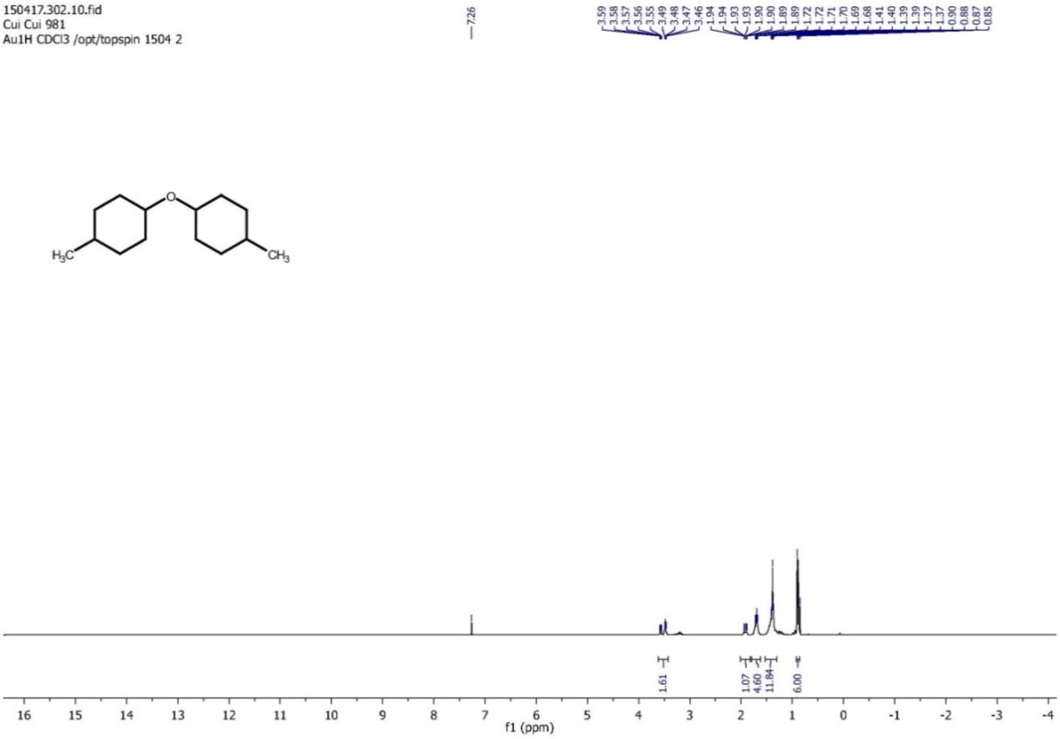

Supplementary Figure 49. ^{1}H NMR (Table 4, entry 2)

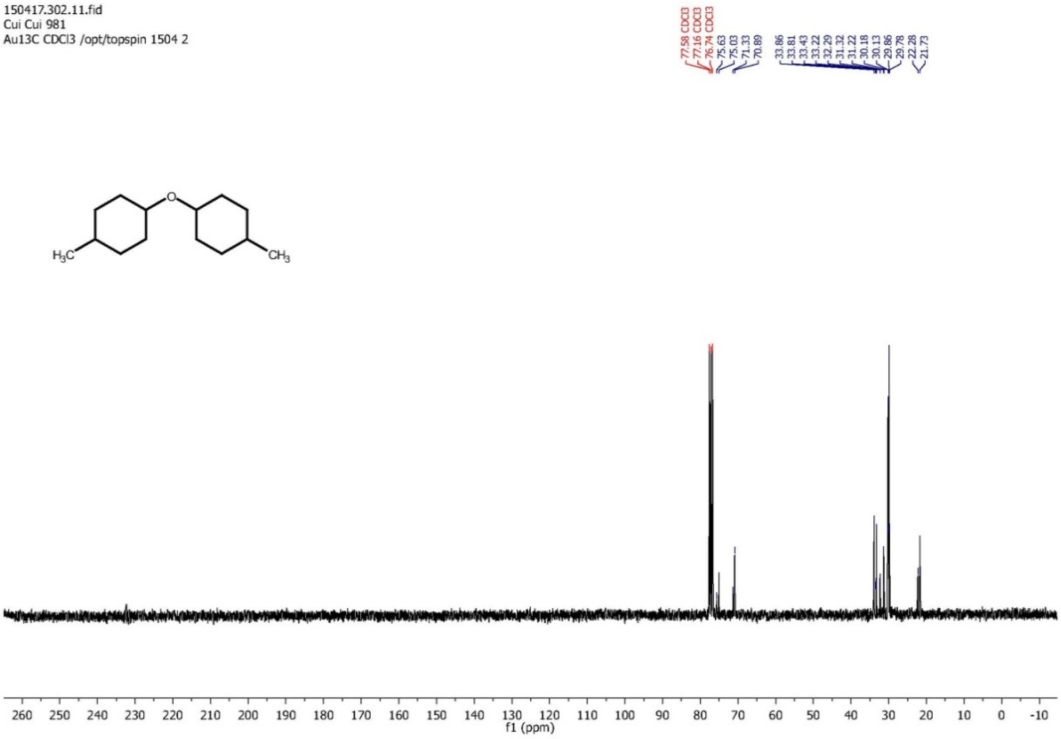

Supplementary Figure 50. ^{13}C NMR (Table 4, entry 2)

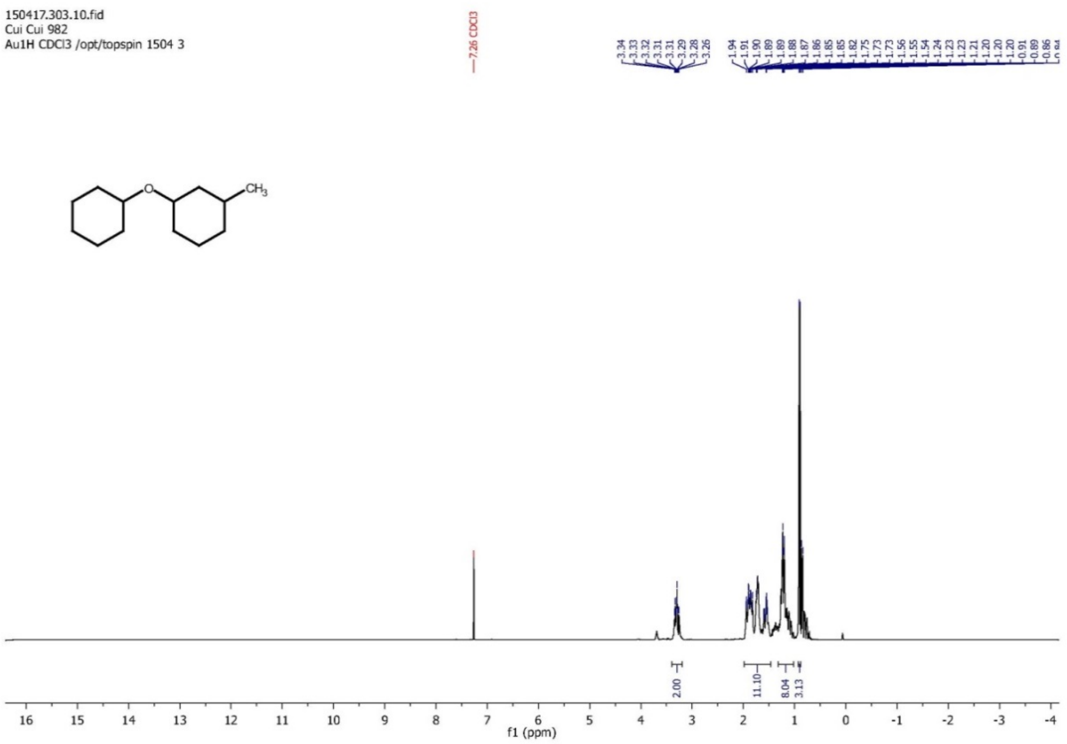

Supplementary Figure 51. ^{1}H NMR (Table 4, entry 3)

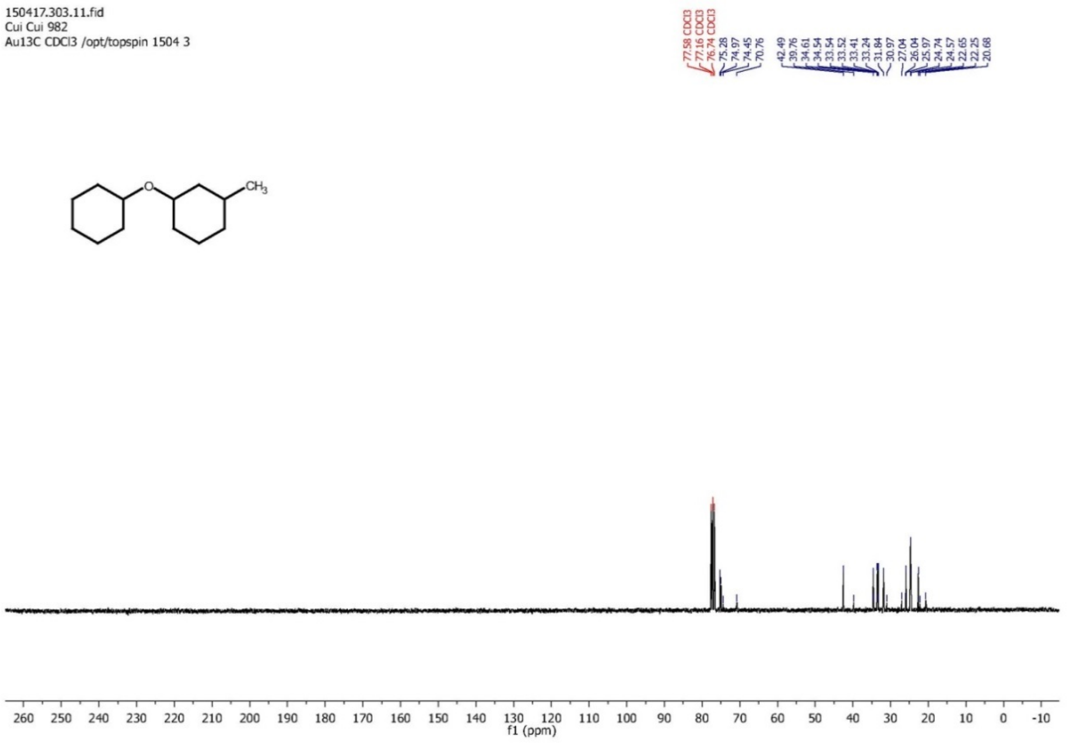

Supplementary Figure 52. ^{13}C NMR (Table 4, entry 3)

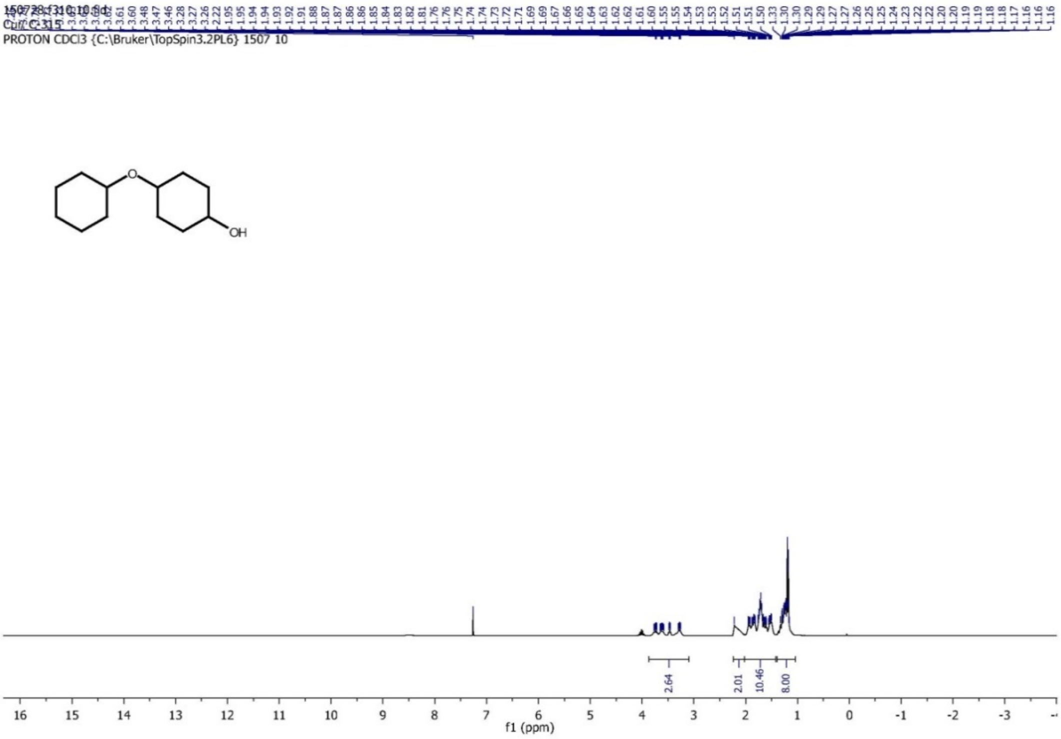

Supplementary Figure 53. ^{1}H NMR (Table 4, entry 4)

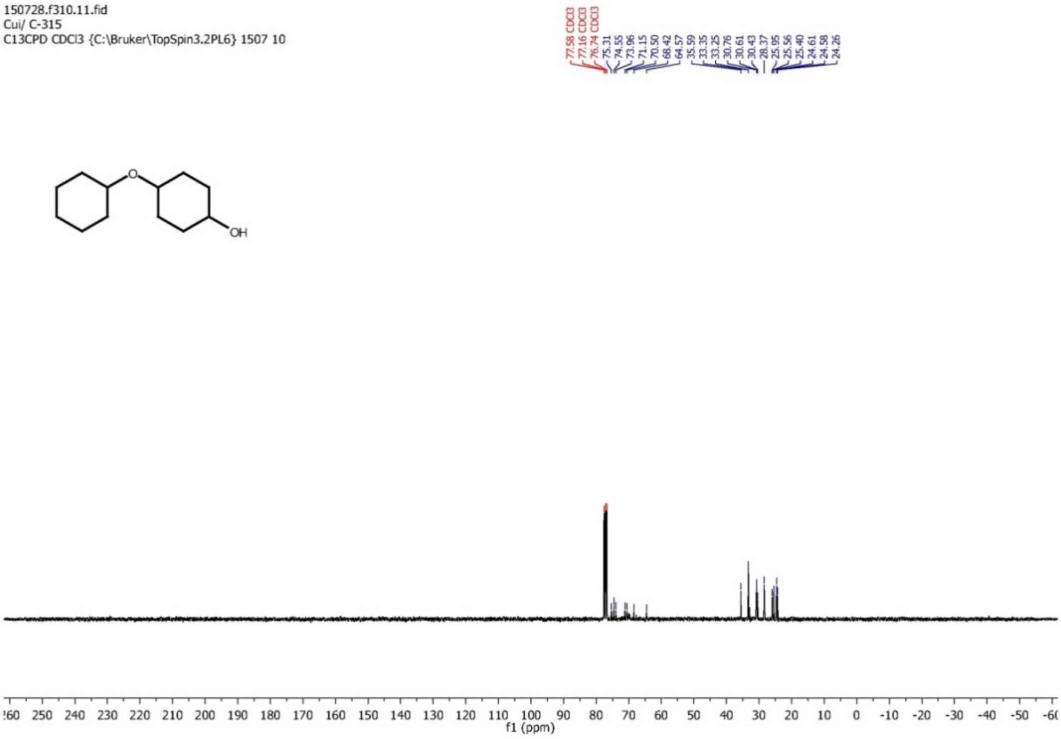

Supplementary Figure 54. ^{13}C NMR (Table 4, entry 4)

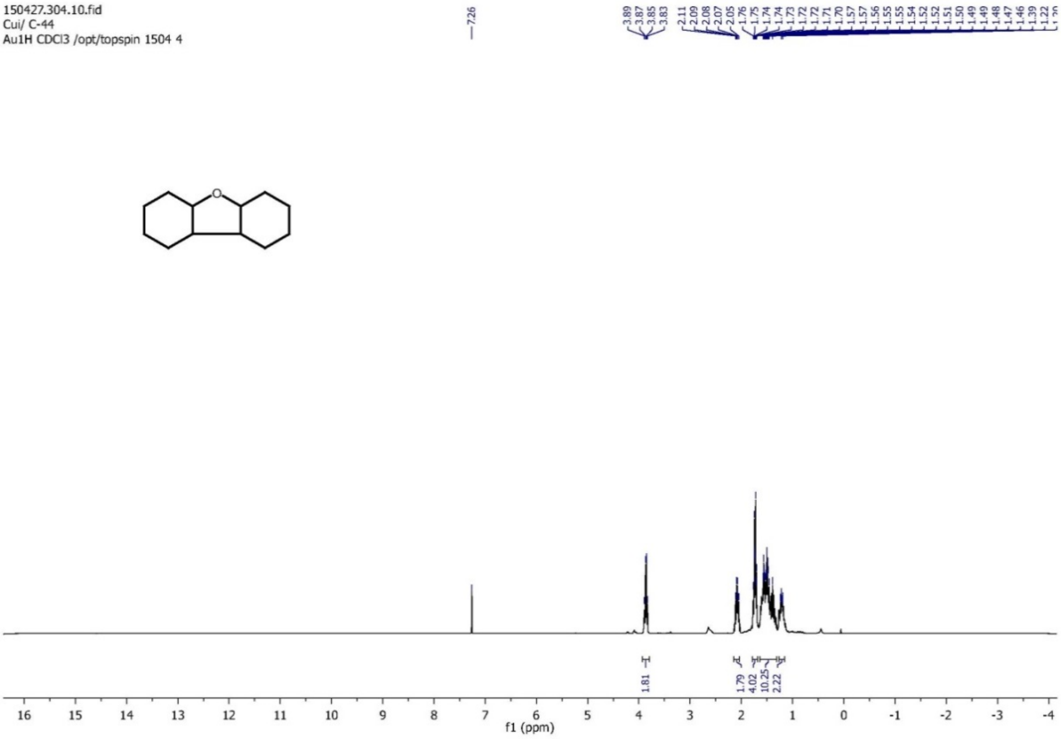

Supplementary Figure 55. ^{1}H NMR (Table 4, entry 5)

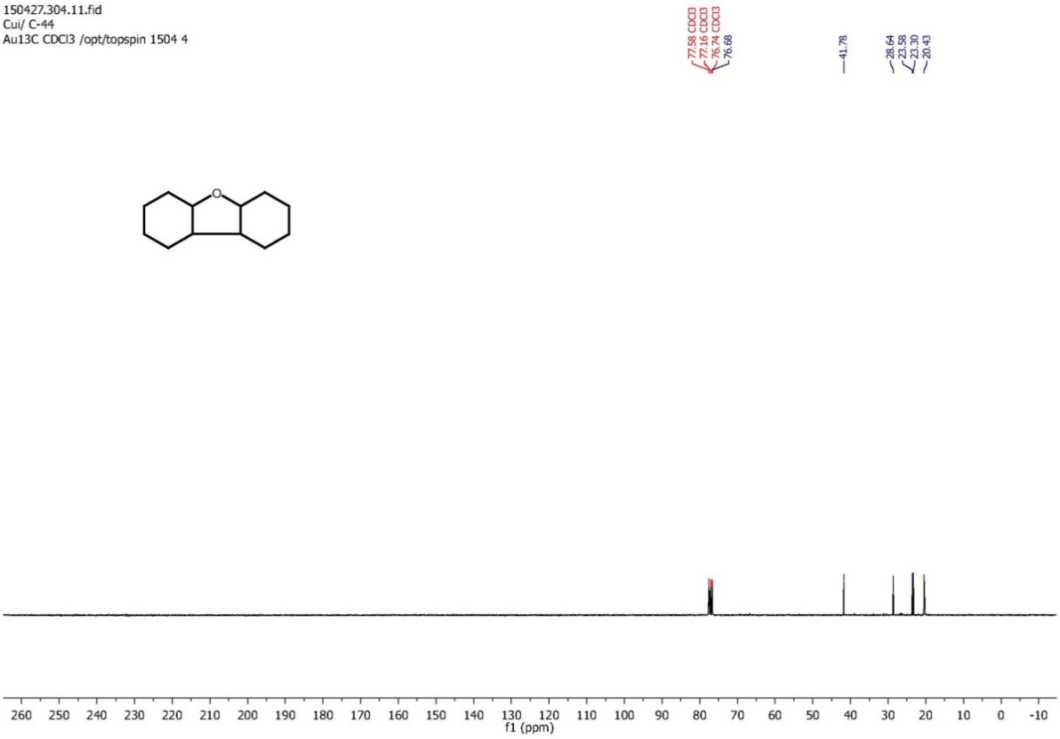

Supplementary Figure 56. ^{13}C NMR (Table 4, entry 5)

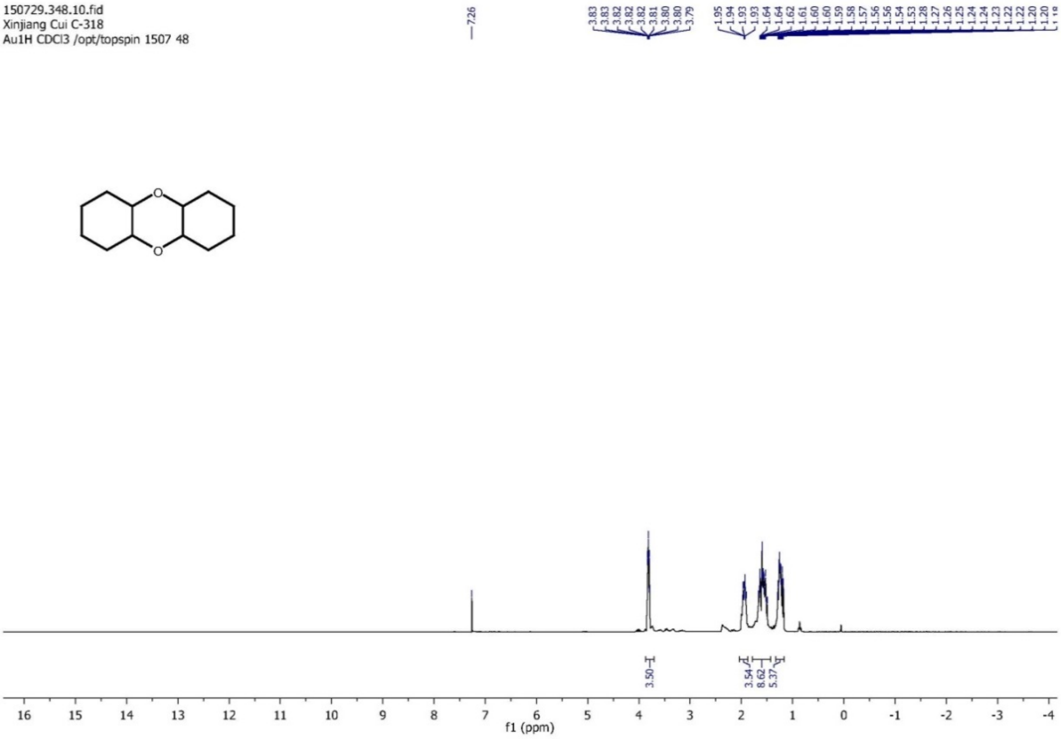

Supplementary Figure 57. ^{1}H NMR (Table 4, entry 6)

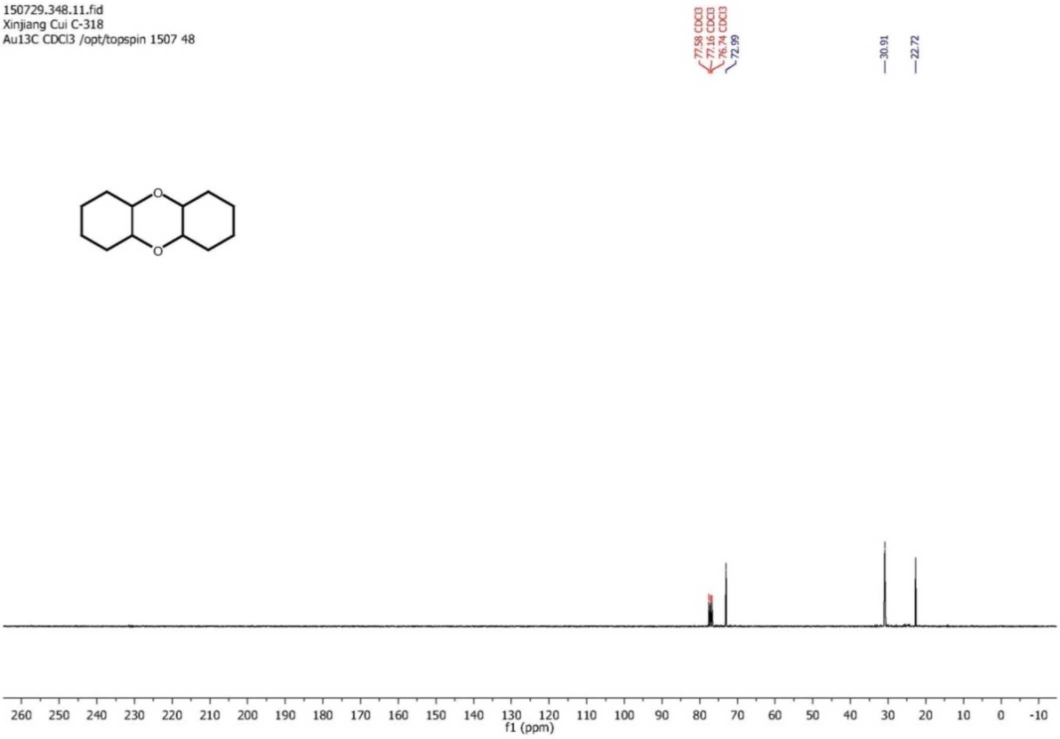

Supplementary Figure 58. ^{13}C NMR (Table 4, entry 6)

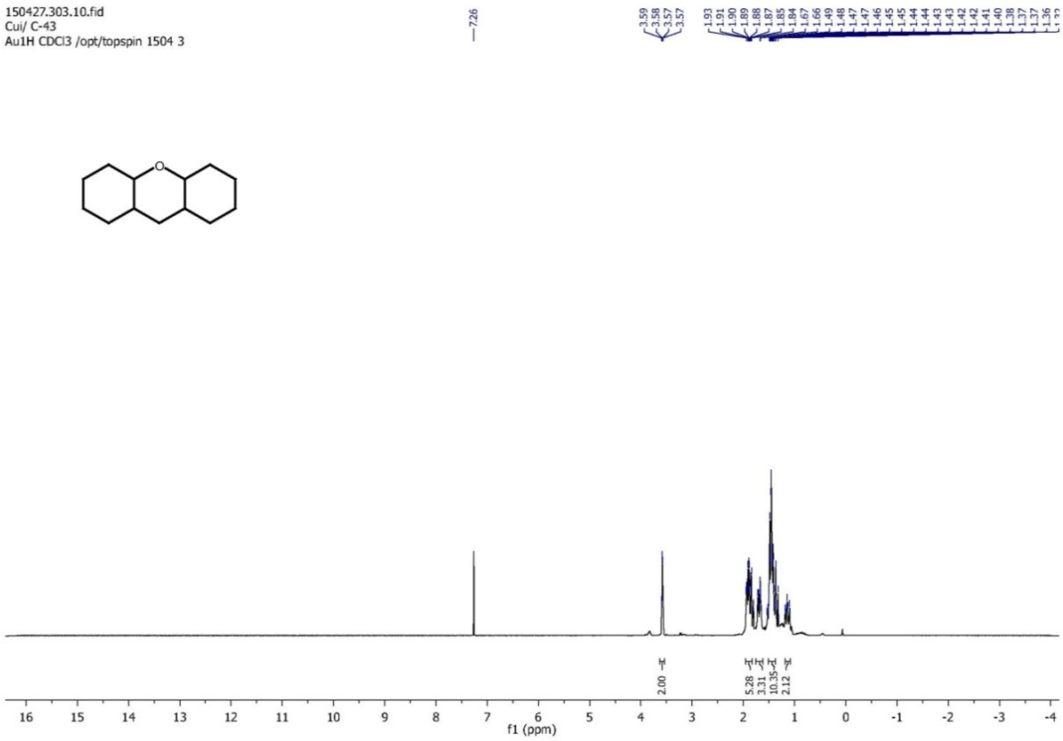

Supplementary Figure 59. ^{1}H NMR (Table 4, entry 7)

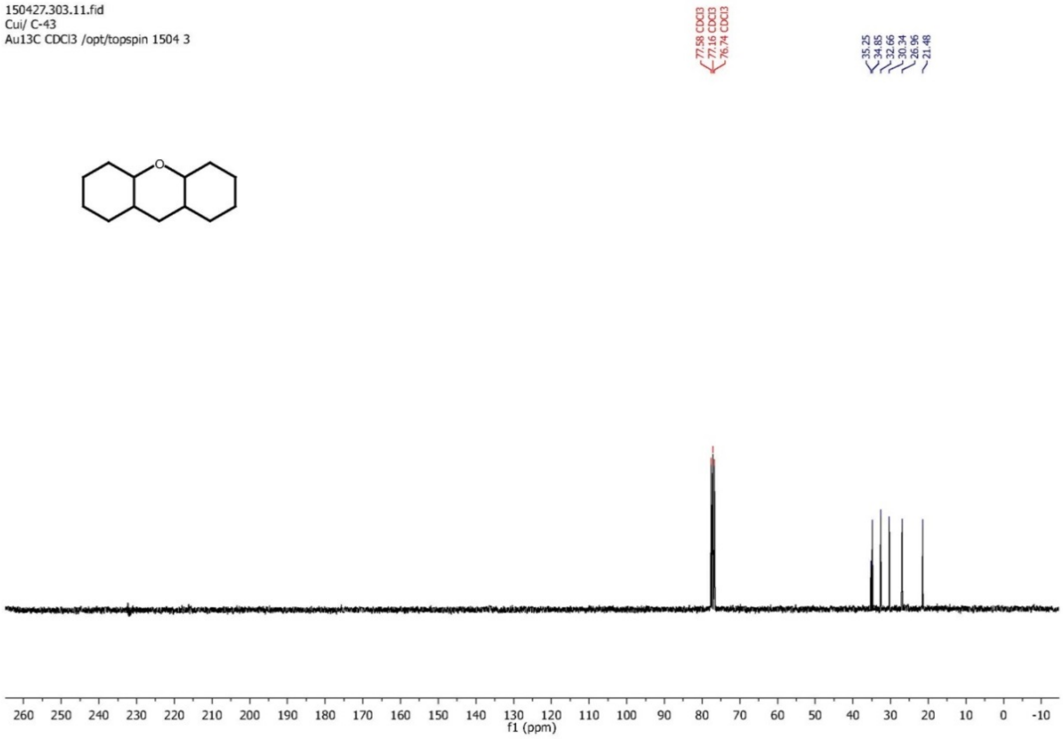

Supplementary Figure 60. ^{13}C NMR (Table 4, entry 7)

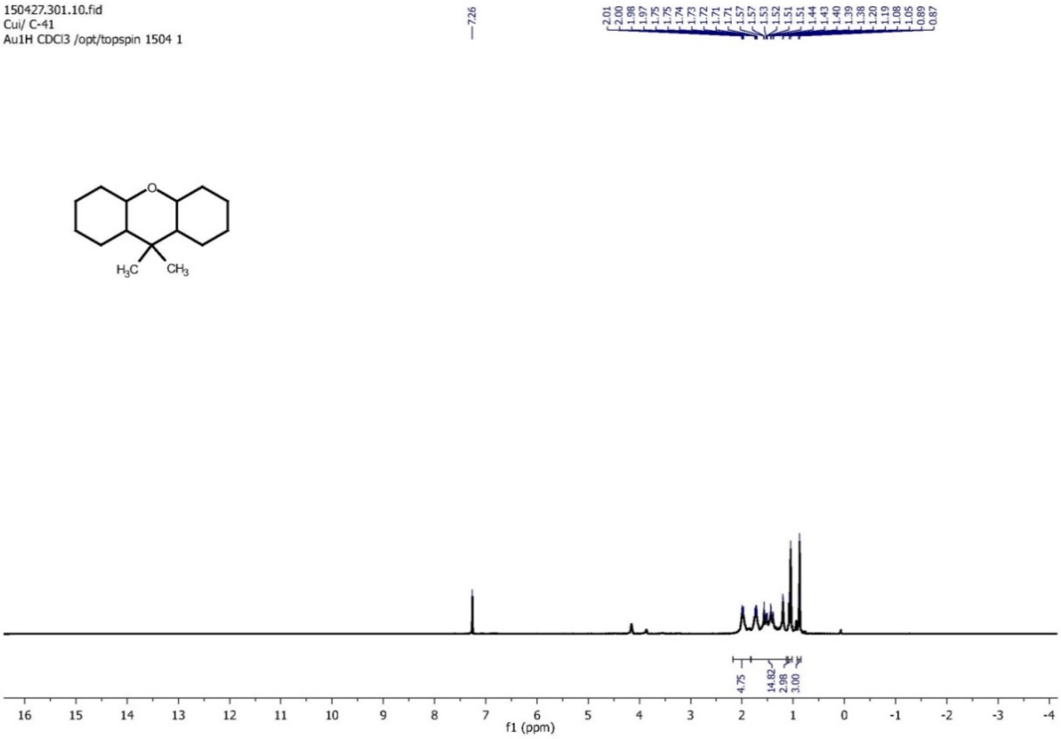

Supplementary Figure 61. ^{1}H NMR (Table 4, entry 8)

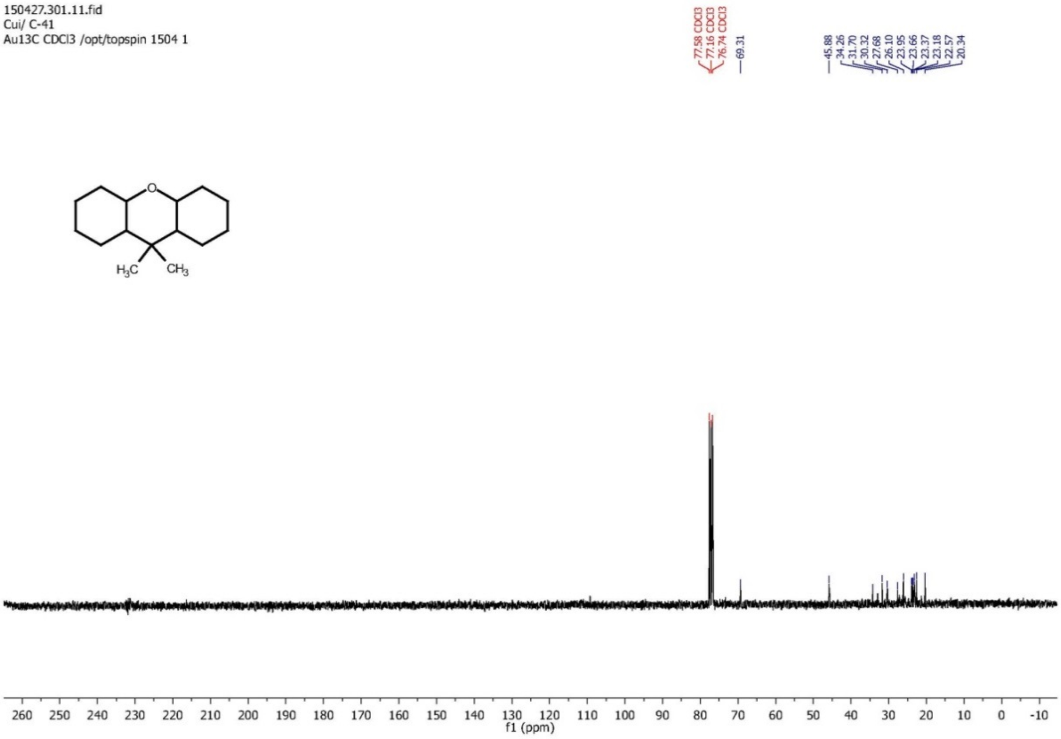

Supplementary Figure 62. ^{13}C NMR (Table 4, entry 8)

Supplementary Table 1 XPS analysis of the prepared catalysts

Catalyst	C	N (EA)	O	Cl
Ru@NDCs-600	94.26	1.72 (0.65)	3.82	0
Ru@NDCs-800	96.44	1.37 (0.51)	2.13	0
Ru@NDCs-800 [a]	97.2	0.66 (0.23)	2.13	0
Ru@NDCs-900	98.32	0.53 (0.40)	1.05	0
Ru@C-800	98.07	0 (0)	1.63	0.12

[a]100 mg ligand used.

Supplementary Table 2 The effect of different solvents in the presence of Ru@NDCs-800[a,b]

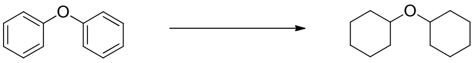

Solvent	Con (%)	Sel (%)
IPA	100	84[c]
Heptane	100	80
THF	100	76
Cyclohexane	100	89
H_2O	100	22

[a] Reaction conditions: 0.5 mmol substrate, 20 mg catalyst, 2 mL isopropanol, 20 bar H_2, 60 °C, 24 h. [b] Results were obtained by GC-FID using dodecane as standard. [c]Isolated yield.

The effect of different solvents on the selectivity was investigated in the catalytic hydrogenation of biphenylether to dicyclohexylether facilitated by Ru@NDCs-800. With water being the only exception, a pronounced effect of the reaction medium was not observed. The selectivities towards the alicyclic product range well between 76% and 89%, whereas cyclohexane afforded the highest value. In contrast, the very low selectivity of 22% obtained in water is caused by undesired hydrogenolysis- and hydrolysis reactions which prevent effective generation of the saturated ether.

Supplementary Table 3 the catalytic activity of the Ru@NDCs in short time[a,b]

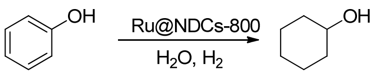

Entry	Catalyst	T (°C)	P (bar)	Y (%)[b]	Sel (%)	TOF
1	Ru@NDCs-600	40	5	20	98	8
2	Ru@NDCs-700	40	5	23	99	9
3	Ru@NDCs-800	40	5	41	99	17
4	Ru@NDCs-900	40	5	6	99	2.5

[a] Reaction conditions: 0.5 mmol substrate, 20 mg catalyst, 2 mL H_2O, 1 h. [b] Results were obtained by GC-FID using dodecane as standard.

According to the results in Table S3, 41% conversion and 99% selectivity were obtained using Ru@NDCs-800 as catalyst. In contrast, Ru@NDCs materials prepared at lower and higher pyrolysis temperature (600 ^oC, 700 ^oC and 900 ^oC) featured lower reactivity.

Supplementary Methods:

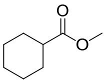

^1H NMR (300 MHz, Chloroform-*d*) δ 3.64 (s, 3H), 2.35 – 2.22 (m, 1H), 1.94 – 1.81 (m, 2H), 1.79 – 1.67 (m, 2H), 1.67 – 1.56 (m, 1H), 1.51 – 1.14 (m, 5H). ^{13}C NMR (75 MHz, $CDCl_3$) δ 176.69, 51.55, 43.23, 29.14, 25.88, 25.57.

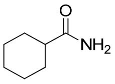

^1H NMR (300 MHz, Chloroform-*d*) δ 6.27 (s, 2H), 2.31 – 2.13 (m, 1H), 1.96 – 1.58 (m, 5H), 1.54 – 1.12 (m, 5H). ^{13}C NMR (75 MHz, $CDCl_3$) δ 180.18, 44.49, 29.63, 25.73, 25.68. HR-MS: Calcd for $C_7H_{13}ON$: 127.09917; Found: 127.09930.

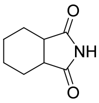

^1H NMR (300 MHz, Chloroform-*d*) δ 8.30 (s, 1H), 2.90 (m, *J* = 4.6, 2.3 Hz, 2H), 1.96 – 1.66 (m, 4H), 1.46 (p, *J* = 3.2 Hz, 4H). ^{13}C NMR (75 MHz, $CDCl_3$) δ 180.05, 100.12, 41.18, 23.89, 21.94. HR-MS: Calcd for $C_8H_{11}N_1O_2$: 153.07843; Found: 153.07809.

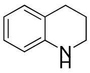

^1H NMR (300 MHz, Chloroform-*d*) δ 6.99 (m, 2H), 6.68 – 6.43 (m, 2H), 3.95 (s, 1H), 3.40 – 3.22 (m, 2H), 2.79 (t, *J* = 6.4 Hz, 2H), 2.10 – 1.87 (m, 2H). ^{13}C NMR (75 MHz, $CDCl_3$) δ 144.40, 129.63, 126.84, 121.83, 117.41, 114.59, 42.10, 27.01, 22.20. Calcd for $C_9H_{11}N_1$: 133.08860; Found: 133.08821.

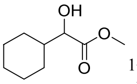

^1H NMR (300 MHz, Chloroform-*d*) δ 4.02 (d, *J* = 3.5 Hz, 1H), 3.78 (s, 3H), 2.49 (s, 1H), 1.83 – 1.56 (m, 5H), 1.43 (m, *J* = 8.1, 4.9, 4.2, 2.3 Hz, 1H), 1.32 – 1.08 (m, 6H). ^{13}C NMR (75 MHz, $CDCl_3$) δ 175.47, 75.02, 52.52, 42.10, 29.22, 26.50, 26.38, 26.14, 26.09. Calcd for $C_9H_{16}O_3$ $(M+H)^+$: 173.11722; Found: 173.11657.

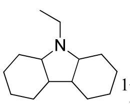

^1H NMR (300 MHz, Chloroform-*d*) δ 2.97 – 2.26 (m, 3H), 2.13 – 0.68 (m, 22H). ^{13}C NMR (75 MHz, $CDCl_3$) δ 71.22, 48.36, 45.62, 41.20, 31.15, 29.15, 25.77, 25.29, 24.19, 22.95, 20.99, 13.32. HR-MS: Calcd for $C_{14}H_2N_1$: 207.19815; Found: 207.19760.

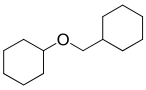

^1H NMR (300 MHz, Chloroform-*d*) δ 3.22 (d, *J* = 6.6 Hz, 2H), 3.20 – 3.11 (m, 1H), 1.98 – 1.44 (m, 11H), 1.34 – 1.10 (m, 8H), 0.88 (m, *J* = 11.4, 3.2 Hz, 2H). ^{13}C NMR (75 MHz, $CDCl_3$) δ 77.67, 76.73, 38.49, 32.47, 30.43, 26.88, 26.06, 24.38. HR-MS: Calcd for $C_{13}H_{24}O$: 196.18217; Found: 196.18212.

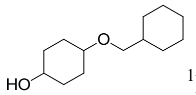

^{1}H NMR (400 MHz, Chloroform-*d*) δ 3.75 – 3.62 (m, 1H), 3.32 (m, *J* = 6.0, 3.0 Hz, 1H), 3.20 (m, *J* = 14.1, 6.6 Hz, 2H), 2.04 – 1.92 (m, 2H), 1.86 – 1.60 (m, 9H), 1.56 – 1.44 (m, 2H), 1.40 – 1.08 (m, 5H), 0.97 – 0.82 (m, 2H). ^{13}C NMR (101 MHz, $CDCl_3$) δ 76.84, 73.88, 69.95, 38.50, 38.47, 32.93, 30.66, 30.52, 30.40, 30.36, 29.50, 27.75, 26.87, 26.84, 26.07, 26.03. HR-MS: Calcd for $C_{13}H_{24}O_2$: 212.17708; Found: 212.17731:

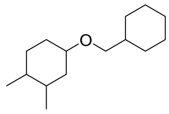

^{1}H NMR (300 MHz, Chloroform-*d*) δ 3.45 – 3.37 (m, 1H), 3.31 (m, *J* = 22.7, 7.1 Hz, 2H), 2.07 – 1.56 (m, 6H), 1.54 – 1.10 (m, 13H), 0.93 – 0.87 (m, 6H). ^{13}C NMR (75 MHz, $CDCl_3$) δ 74.02, 71.31, 38.29, 35.85, 34.97, 33.65, 32.52, 31.59, 30.97, 30.93, 30.39, 30.29, 29.65, 29.62, 29.58, 26.00, 21.98, 20.38. HR-MS: Calcd for $C_{15}H_{28}O_1$: 224.21355; Found: 224.21347.

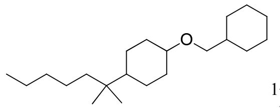

^{1}H NMR (300 MHz, Chloroform-*d*) δ 3.50 – 3.43 (m, 1H), 3.23 (d, *J* = 6.6 Hz, 1H), 3.14 (d, *J* = 6.6 Hz, 1H), 2.09 – 1.90 (m, 2H), 1.83 – 1.59 (m, 7H), 1.38 – 1.03 (m, 11H), 0.94 (d, *J* = 15.9 Hz, 18H). ^{13}C NMR (75 MHz, $CDCl_3$) δ 78.73, 74.22, 73.47, 72.80, 51.71, 51.49, 49.09, 48.62, 38.49, 38.45, 37.11, 36.84, 33.00, 32.43, 32.40, 32.37, 30.83, 30.44, 27.00, 26.94, 26.91, 26.88, 26.10, 26.06, 25.49, 21.25. Calcd for $C_{21}H_{40}O([M+Na]^+)$: 331.29714; Found: 331.29734.

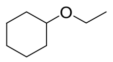

^{1}H NMR (300 MHz, Chloroform-*d*) δ 3.50 (q, *J* = 7.0 Hz, 2H), 3.28 – 3.16 (m, 1H), 2.00 – 1.86 (m, 2H), 1.81 – 1.63 (m, 2H), 1.53 (m, 1H), 1.35 – 1.03 (m, 8H). ^{13}C NMR (75 MHz, $CDCl_3$) δ 63.08, 35.67, 32.58, 26.00, 24.47, 15.88.

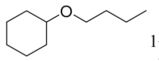

^{1}H NMR (300 MHz, Chloroform-*d*) δ 3.42 (m, 2H), 3.27 – 3.10 (m, 1H), 1.89 (m, 2H), 1.78 – 1.65 (m, 2H), 1.59 – 1.46 (m, 3H), 1.36 – 1.16 (m, 7H), 0.90 (t, *J* = 7.3 Hz, 3H). ^{13}C NMR (75 MHz, $CDCl_3$) δ 67.73, 32.50, 32.45, 26.01, 24.40, 19.55, 14.05.

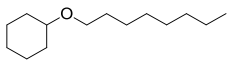

^{1}H NMR (300 MHz, Chloroform-*d*) δ 3.42 (t, *J* = 6.8 Hz, 2H), 3.26 – 3.13 (m, 1H), 1.90 (q, *J* = 1.3 Hz, 2H), 1.72 (q, *J* = 2.0, 1.3 Hz, 2H), 1.62 – 1.45 (m, 3H), 1.40 – 1.08 (m, 15H), 0.95 – 0.81 (m, 3H). ^{13}C NMR (75 MHz, $CDCl_3$) δ 68.11, 32.55, 32.00, 30.40, 29.63, 29.45, 26.41, 26.03, 24.45, 22.81, 14.25. HR-MS: Calcd for $C_{14}H_{28}O$: 212.21347; Found: 212.21302.

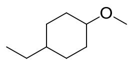

^{1}H NMR (300 MHz, Chloroform-*d*) δ 3.41 – 3.36 (m, 1H), 3.34 (s, 1H), 3.30 (s, 2H), 2.10 – 1.94 (m, 1H), 1.89 – 1.74 (m, 2H), 1.53 – 1.34 (m, 3H), 1.30 – 1.15 (m, 5H), 0.90 – 0.84 (m, 3H). ^{13}C NMR (75 MHz, $CDCl_3$) δ 80.05, 76.02, 55.74, 55.68, 38.92, 38.47, 35.76, 31.96, 31.03, 31.00, 29.51, 29.15, 29.07, 27.05, 11.85, 11.72.

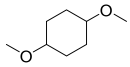

^{1}H NMR (300 MHz, Chloroform-*d*) δ 3.33 (s, 1H), 3.31 (s, 5H), 3.29 – 3.20 (m, 2H), 2.09 – 1.86 (m, 1H), 1.83 – 1.65 (m, 4H), 1.64 – 1.44 (m, 3H), 1.38 – 1.15 (m, 1H). ^{13}C NMR (75 MHz, $CDCl_3$) δ 78.20, 76.52, 55.60, 28.78, 27.13.

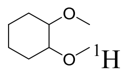 ^{1}H NMR (300 MHz, Chloroform-*d*) δ 3.42 (s, 1H), 3.39 (d, *J* = 2.6 Hz, 2H), 3.37 (s, 5H), 1.93 – 1.77 (m, 2H), 1.59 (m, 2H), 1.53 – 1.40 (m, 2H), 1.28 (m, 2H). ^{13}C NMR (75 MHz, $CDCl_3$) δ 82.53, 78.76, 57.14, 56.34, 29.28, 26.68, 23.58, 21.98.

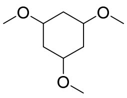 ^{1}H NMR (300 MHz, Chloroform-*d*) δ 3.36 (s, 10H), 3.22 – 3.04 (m, 3H), 2.49 – 2.39 (m, 3H), 1.26 – 1.02 (m, 4H). ^{13}C NMR (75 MHz, $CDCl_3$) δ 74.78, 56.19, 56.16, 37.47, 35.02. HR-MS: Calcd for $C_9H_{18}O_3$: 174.12505; Found: 174.12506.

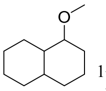 ^{1}H NMR (300 MHz, Chloroform-*d*) δ 3.32 (s, 3H), 3.16 (m, 1H), 2.08 – 1.97 (m, 1H), 1.83 – 1.61 (m, 4H), 1.61 – 1.46 (m, 4H), 1.46 – 1.12 (m, 8H). ^{13}C NMR (75 MHz, $CDCl_3$) δ 82.53, 55.63, 39.47, 35.79, 32.13, 26.56, 26.42, 25.06, 24.56, 21.72, 19.35.

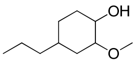 1H NMR (300 MHz, Chloroform-d) δ 3.44 – 3.32 (m, 3H), 2.12 (s, 1H), 2.04 – 1.88 (m, 1H), 1.84 – 1.46 (m, 3H), 1.46 – 1.07 (m, 10H), 0.94 – 0.82 (m, 4H). 13C NMR (75 MHz, CDCl3) δ 84.86, 81.02, 74.20, 65.89, 56.49, 55.89, 39.22, 38.98, 35.80, 35.74, 34.97, 32.03, 31.39, 30.61, 29.90, 25.78, 20.31, 20.07, 14.49, 14.44.

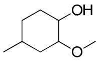 1H NMR (300 MHz, Chloroform-d) δ 4.11 – 3.90 (m, 1H), 3.37 (m, 2H), 2.32 (m, 2H), 2.11 – 1.82 (m, 1H), 1.78 – 1.55 (m, 1H), 1.52 – 1.12 (m, 7H), 1.02 – 0.80 (m, 3H). 13C NMR (75 MHz, CDCl3) δ 84.68, 80.90, 73.91, 65.56, 64.59, 56.42, 55.86, 36.99, 35.66, 33.93, 33.44, 32.70, 31.56, 31.19, 31.06, 29.93, 29.10, 27.77, 25.39, 22.30, 21.99.

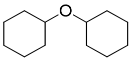 ^{1}H NMR (300 MHz, Chloroform-*d*) δ 3.31 (m, 2H), 2.05 – 1.62 (m, 8H), 1.62 – 1.44 (m, 2H), 1.22 (m, 10H). ^{13}C NMR (75 MHz, $CDCl_3$) δ 74.85, 33.51, 25.98, 24.76.

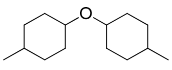 ^{1}H NMR (300 MHz, Chloroform-*d*) δ 3.53 (m, 2H), 1.92 (m, 1H), 1.70 (m, 5H), 1.39 (m, 12H), 0.92 – 0.86 (m, 6H). ^{13}C NMR (75 MHz, $CDCl_3$) δ 75.63, 75.03, 71.33, 70.89, 33.86, 33.81, 33.43, 33.22, 32.29, 31.32, 31.22, 30.18, 30.13, 29.86, 29.78, 22.28, 21.73. HR-MS: Calcd for $C_{14}H_{26}O$: 210.19782; Found: 210.19800.

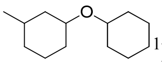 ^{1}H NMR (300 MHz, Chloroform-*d*) δ 3.40 – 3.19 (m, 2H), 1.99 – 1.47 (m, 11H), 1.32 – 1.02 (m, 8H), 0.90 (d, *J* = 6.6 Hz, 3H). ^{13}C NMR (75 MHz, $CDCl_3$) δ 75.28, 74.97, 74.45, 70.76, 42.49, 39.76, 34.61, 34.54, 33.54, 33.52, 33.41, 33.24, 31.84, 30.97, 27.04, 26.04, 25.97, 24.74, 24.57, 22.65, 22.25, 20.68. HR-MS: Calcd for $C_{13}H_{24}O$: 196.18217; Found: 196.18232.

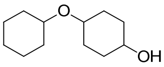 ^{1}H NMR (300 MHz, Chloroform-*d*) δ 3.86 – 3.09 (m, 3H), 2.22 (s, 2H), 2.02 – 1.42 (m, 10H), 1.40 – 1.04 (m, 8H). ^{13}C NMR (75 MHz, $CDCl_3$) δ 75.31, 74.55, 73.96, 71.15, 70.50, 68.42, 64.57, 35.59, 33.35, 33.25, 30.76, 30.61, 30.43, 28.37, 25.95, 25.56, 25.40, 24.61, 24.58, 24.26. HR-MS: Calcd for $C_{12}H_{22}O_2$: 198:16143; Found: 198:16148.

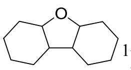 ^{1}H NMR (300 MHz, Chloroform-*d*) δ 3.86 (q, *J* = 6.1 Hz, 2H), 2.14 – 2.03 (m, 2H), 1.78 – 1.69 (m, 4H), 1.64 – 1.31 (m, 10H), 1.26 – 1.16 (m, 2H). ^{13}C NMR (75 MHz, $CDCl_3$) δ 76.68, 41.78, 28.64, 23.58, 23.30, 20.43. HR-MS: Calcd for $C_{12}H_{20}O$: 180.15087; Found: 180.15109.

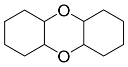
^{1}H NMR (300 MHz, Chloroform-*d*) δ 3.82 (m, 4H), 2.04 – 1.88 (m, 3H), 1.79 – 1.43 (m, 8H), 1.33 – 1.16 (m, 5H). ^{13}C NMR (75 MHz, $CDCl_3$) δ 77.58, 77.16, 76.74, 72.99, 30.91, 22.72.

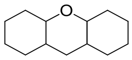
^{1}H NMR (300 MHz, Chloroform-*d*) δ 3.58 (q, *J* = 2.7 Hz, 2H), 1.96 – 1.83 (m, 5H), 1.76 – 1.62 (m, 3H), 1.51 – 1.37 (m, 10H), 1.14 (m, 2H). ^{13}C NMR (75 MHz, $CDCl_3$) δ 35.25, 34.85, 32.66, 30.34, 26.96, 21.48. HR-MS: Calcd for $C_{13}H_{22}O$: 194.16652; Found: 194.16689.

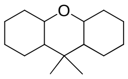
^{1}H NMR (300 MHz, Chloroform-*d*) δ 1.99 (m, 5H), 1.82 – 1.13 (m, 15H), 1.06 (d, *J* = 9.1 Hz, 3H), 0.88 (d, *J* = 4.9 Hz, 3H). ^{13}C NMR (75 MHz, $CDCl_3$) δ 69.31, 45.88, 34.26, 31.70, 30.32, 27.68, 26.10, 23.95, 23.66, 23.37, 23.18, 22.57, 20.34. HR-MS: Calcd for $C_{15}H_{26}O$: 222.19782; Found: 222.19735.
